# SARS-CoV-2 vaccination in the first year after allogeneic hematopoietic cell transplant: a prospective, multicentre, observational study

**DOI:** 10.1016/j.eclinm.2023.101983

**Published:** 2023-04-27

**Authors:** Joshua A. Hill, Michael J. Martens, Jo-Anne H. Young, Kavita Bhavsar, Jianqun Kou, Min Chen, Lik Wee Lee, Aliyah Baluch, Madhav V. Dhodapkar, Ryotaro Nakamura, Kristin Peyton, Zainab Shahid, Paul Armistead, Peter Westervelt, John McCarty, Joseph McGuirk, Mehdi Hamadani, Susan DeWolf, Kinga Hosszu, Elad Sharon, Ashley Spahn, Amir A. Toor, Stephanie Waldvogel, Lee M. Greenberger, Jeffery J. Auletta, Mary M. Horowitz, Marcie L. Riches, Miguel-Angel Perales

**Affiliations:** a Vaccine and Infectious Disease, Fred Hutchinson Cancer Center, Seattle, WA, USA; b Department of Medicine, University of Washington, Seattle, WA, USA; c Center for International Blood and Marrow Transplantation Research, Medical College of Wisconsin, Milwaukee, WI, USA; d Division of Biostatistics, Medical College of Wisconsin, Milwaukee, WI, USA; e University of Minnesota, Minneapolis, MN, USA; f Adaptive Biotechnologies Corp, Seattle, WA, USA; g H. Lee Moffitt Cancer Center and Research Institute, Tampa, FL, USA; h Emory University – School of Medicine, Atlanta, GA, USA; i City of Hope, Duarte, CA, USA; j The Emmes Company, Rockville, MD, USA; k Memorial Sloan Kettering Cancer Center, New York, NY, USA; l University of North Carolina Medical Center, Chapel Hill, NC, USA; m Barnes-Jewish Hospital, Washington University, St. Louis, MO, USA; n Virginia Commonwealth University, Richmond, VA, USA; o University of Kansas, Lawrence, KS, USA; p Medical College of Wisconsin, Milwaukee, WI, USA; q National Cancer Institute, Bethesda, MD, USA; r National Marrow Donor Program/Center for International Blood and Marrow Transplant Research, Minneapolis, MN, USA; s The Leukemia and Lymphoma Society, Rye Brook, New York, NY, USA; t Nationwide Children's Hospital, Columbus, OH, USA; u Weil Cornell Medical College, New York, NY, USA

**Keywords:** SARS-CoV-2, Covid-19, Vaccine, Transplant, Hematopoietic cell transplant

## Abstract

**Background:**

The optimal timing for SARS-CoV-2 vaccines within the first year after allogeneic hematopoietic cell transplant (HCT) is poorly understood.

**Methods:**

We conducted a prospective, multicentre, observational study of allogeneic HCT recipients who initiated SARS-CoV-2 vaccinations within 12 months of HCT. Participants were enrolled at 22 academic cancer centers across the United States. Participants of any age who were planning to receive a first post-HCT SARS-CoV-2 vaccine within 12 months of HCT were eligible. We obtained blood prior to and after each vaccine dose for up to four vaccine doses, with an end-of-study sample seven to nine months after enrollment. We tested for SARS-CoV-2 spike protein (anti-S) IgG; nucleocapsid protein (anti-N) IgG; neutralizing antibodies for Wuhan D614G, Delta B.1.617.2, and Omicron B.1.1.529 strains; and SARS-CoV-2-specific T-cell receptors (TCRs). The primary outcome was a comparison of anti-S IgG titers at the post-V2 time point in participants initiating vaccinations <4 months versus 4–12 months after HCT using a propensity-adjusted analysis. We also evaluated factors associated with high-level anti-S IgG titers (≥2403 U/mL) in logistic regression models.

**Findings:**

Between April 22, 2021 and November 17, 2021, 175 allogeneic HCT recipients were enrolled in the study, of whom all but one received mRNA SARS-CoV-2 vaccines. SARS-CoV-2 anti-S IgG titers, neutralizing antibody titers, and TCR breadth and depth did not significantly differ at all tested time points following the second vaccination among those initiating vaccinations <4 months versus 4–12 months after HCT. Anti-S IgG ≥2403 U/mL correlated with neutralizing antibody levels similar to those observed in a prior study of non-immunocompromised individuals, and 57% of participants achieved anti-S IgG ≥2403 U/mL at the end-of-study time point. In models adjusted for SARS-CoV-2 infection pre-enrollment, SARS-CoV-2 vaccination pre-HCT, CD19+ B-cell count, CD4+ T-cell count, and age (as applicable to the model), vaccine initiation timing was not associated with high-level anti-S IgG titers at the post-V2, post-V3, or end-of-study time points. Notably, prior graft-versus-host-disease (GVHD) or use of immunosuppressive medications were not associated with high-level anti-S IgG titers. Grade ≥3 vaccine-associated adverse events were infrequent.

**Interpretation:**

These data support starting mRNA SARS-CoV-2 vaccination three months after HCT, irrespective of concurrent GVHD or use of immunosuppressive medications. This is one of the largest prospective analyses of vaccination for any pathogen within the first year after allogeneic HCT and supports current guidelines for SARS-CoV-2 vaccination starting three months post-HCT. Additionally, there are few studies of mRNA vaccine formulations for other pathogens in HCT recipients, and these data provide encouraging proof-of-concept for the utility of early vaccination targeting additional pathogens with mRNA vaccine platforms.

**Funding:**

National Marrow Donor Program, Leukemia and Lymphoma Society, Multiple Myeloma Research Foundation, Novartis, LabCorp, American Society for Transplantation and Cellular Therapy, 10.13039/100016796Adaptive Biotechnologies, and the 10.13039/100000002National Institutes of Health.

**Research in context**

**Evidence before this study**

To assess previous research pertaining to SARS-CoV-2 vaccine responses in allogeneic hematopoietic cell transplant (HCT) recipients, we conducted a non-systematic PubMed search using a combination of terms including, but not limited to, ‘SARS-CoV-2’, ‘Covid-19’, ‘vaccine’, ‘transplant’, and ‘hematopoietic cell transplant’. The search included clinical trials, clinical observations, and treatment guidelines (with no start date up to December 16, 2022). The optimal timing for SARS-CoV-2 vaccines within the first year after allogeneic HCT remains poorly understood. Current guidelines suggest initiating the vaccine series as early as three months based on historical experience with vaccines targeting other pathogens, none of which used the mRNA platform. Initial studies of SARS-CoV-2 vaccination in allogeneic HCT recipients demonstrated lower immunogenicity compared to healthy individuals, particularly when given sooner after HCT. However, most studies are limited by small sample sizes, with a minority of individuals vaccinated within the first three to twelve months after HCT. Many studies also lack data pertaining to T-cell responses or neutralizing antibodies, which are better surrogates for protection from severe COVID-19. Thus, there are insufficient data to inform whether vaccination starting at three months after HCT is as effective as delayed vaccination.

**Added value of this study**

To address this knowledge gap that directly impacts clinical practice, the Center for International Blood and Marrow Transplant Research (CIBMTR) and Blood and Marrow Transplant Clinical Trials Network (BMT CTN) conducted a multi-center, prospective, observational study of the safety and immunogenicity of SARS-CoV-2 vaccination within 12 months after allogeneic HCT. In a cohort of 175 allogeneic HCT recipients from 22 cancer centers, we demonstrate that SARS-CoV-2 anti-S IgG titers, neutralizing antibody titers, and SARS-CoV-2 T-cell receptor repertoire breadth and depth were similar after two and three vaccines among those initiating vaccinations <4 months versus 4–12 months after HCT. Grade ≥3 vaccine-associated adverse events were infrequent.

**Implications of all the available evidence**

This study is one of the largest prospective analyses of early vaccination for any pathogen after allogeneic HCT and the first to provide robust evidence in support of current guidelines for SARS-CoV-2 vaccination starting three months post-HCT. Additionally, there are few studies of mRNA vaccine formulations for other pathogens in HCT recipients, so these data provide encouraging proof-of-concept for the utility of early post-HCT vaccines targeting additional pathogens with mRNA vaccine platforms. We conclude that the SARS-CoV-2 mRNA vaccination series should be routinely initiated between three to four months after allogeneic HCT, irrespective of concurrent graft-versus-host disease or use of immunosuppressive medications.

## Introduction

Individuals with hematologic malignancies, and particularly recipients of allogeneic hematopoietic cell transplant (HCT), have a high risk for morbidity and mortality from infection with severe acute respiratory syndrome coronavirus 2 (SARS-CoV-2), the cause of coronavirus disease 2019 (Covid-19).^1^, ^2^, ^3^, ^4^ Initial studies in this patient population demonstrated that up to 30% of allogeneic HCT recipients died within 4–6 weeks after infection with SARS-CoV-2, and infection within the first 12 months after HCT was associated with an increased risk of overall mortality.^5^^,^^6^ With nearly 12,000 allogeneic HCTs performed annually in the United States alone,^7^ there is a large and recurring group of people at high-risk for severe Covid-19.

The pivotal SARS-CoV-2 vaccine clinical trials excluded HCT recipients. As a result, no data were available from early trials to guide vaccination strategies in this high-risk population. Initial studies of SARS-CoV-2 vaccination in cancer patients demonstrated substantially lower immunogenicity in those with hematologic malignancies receiving chemotherapy.^8^, ^9^, ^10^, ^11^, ^12^ Studies in allogeneic HCT recipients soon followed but were limited by small sample sizes, with few individuals vaccinated within the first three to twelve months after HCT.^13^, ^14^, ^15^, ^16^, ^17^, ^18^, ^19^, ^20^, ^21^, ^22^ Furthermore, most studies lack data pertaining to T-cell responses or neutralizing antibodies, which are better surrogates for protection from severe Covid-19.^23^^,^^24^ Nonetheless, these results indicated that vaccination was safe but had relatively low immunogenicity after allogeneic HCT, with limited data suggesting lower responses when vaccination occurred early after HCT.

Although immune responses to standard vaccines following HCT are known to be diminished, guidelines recommend administering inactivated vaccines as soon as three to six months.^25^, ^26^, ^27^, ^28^, ^29^ Based on these historical data, preliminary guidelines recommended consideration for SARS-CoV-2 vaccination as early as three months post-HCT.^30^^,^^31^ However, uncertainty remains about the optimal timing for SARS-CoV-2 vaccines after allogeneic HCT, and there are insufficient data to inform whether vaccination starting at three months after HCT is as effective as delayed vaccination. To address this, the Center for International Blood and Marrow Transplant Research (CIBMTR) and Blood and Marrow Transplant Clinical Trials Network (BMT CTN) conducted a multi-center, prospective, observational study of the safety and immunogenicity of SARS-CoV-2 vaccination within 12 months after allogeneic HCT.

## Methods

### Participants and study design

We prospectively enrolled patients of any age who underwent an allogeneic HCT and were planning to receive a first post-HCT SARS-CoV-2 vaccine within 12 months of HCT. Type of SARS-CoV-2 vaccine, number of doses, and timing post-HCT were at the discretion of participating centers. The study (CIBMTR SC21-07/BMT CTN 2101) opened to enrollment in April 2021 and was approved by the institutional review board of the National Marrow Donor Program. All participants provided written or oral informed consent as appropriate. This study follows the Strengthening the Reporting of Observational Studies in Epidemiology (STROBE) reporting guideline for observational studies.

### Procedures

Blood was obtained at five time points. Samples were collected within pre-specified windows of two weeks prior to first vaccination (pre-V1), at least 3 weeks after first vaccination and within one week prior to second vaccination (post-V1), one to five weeks after second and third vaccination (post-V2 and post-V3, respectively), and seven to nine months after enrollment (end-of-study) (Fig. S1). Data collection is detailed in the Supplement.

### Testing

#### Binding and neutralizing antibodies

We tested serum for semiquantitative total IgG to the SARS-CoV-2 spike protein (S) receptor-binding domain with the Roche Elecsys Anti-SARS-CoV-2 S assay (anti-S IgG), qualitative detection of high-affinity antibodies to SARS-CoV-2 nucleocapsid (N) protein (anti-N IgG), and neutralizing antibodies for Wuhan D614G, Delta B.1.617.2, and Omicron B.1.1.529 strains, at LabCorp (Burlington, NC) as detailed in the Supplement. Anti-S IgG values >0.8 units per milliliter (U/mL) and neutralizing titers ≥40 inhibitory dose (ID50) (reciprocal of the sample dilution required to reduce relative luminescence units by 50%) were considered positive as previously described.^32^ The upper limit of quantitation for anti-S IgG was 2500 U/mL. Titers below the limit of detection (LOD) were assigned a value of one-half the LOD. All time points were tested for anti-S IgG; only baseline samples were tested for anti-N IgG. Neutralizing antibodies were tested at the pre-V1, post-V2, and post-V3 or end-of-study (based on sample availability) time points in a subgroup of 60 chronologically enrolled participants.

#### SARS-CoV-2-specific T-cells

We performed T-cell receptor (TCR) variable beta chain immunosequencing of genomic DNA from peripheral blood mononuclear cells (PBMCs) using the ImmunoSEQ Assay (Adaptive Biotechnologies, Seattle, WA) to quantify the absolute abundance of unique SARS-CoV-2–specific TCRs as previously described.^33^, ^34^, ^35^ We quantified SARS-CoV-2 TCR breadth, defined as the proportion of total unique TCRs associated with SARS-CoV-2, and depth, defined as the extent to which SARS-CoV-2-associated TCRs expand. Samples were classified as positive, negative, or “no call” (representing samples with insufficient TCR rearrangements) using the T-Detect classifier based on breadth and depth compared to a reference population of individuals with prior SARS-CoV-2 infection. Testing was performed in the same subgroup of 60 individuals tested for neutralizing antibodies.

#### Multiparametric flow cytometric analysis

Cryopreserved PBMCs from pre-V1 samples were tested by flow cytometry to delineate the percentage and absolute counts of CD19+ B-cells and CD4+ T-cells as detailed in the Supplement.

### Statistical analysis

The primary objective was to compare the immunogenicity of SARS-CoV-2 vaccines in patients starting <4 months versus 4–12 months after allogeneic HCT. We hypothesized that SARS-CoV-2 vaccines would be safe and immunogenic in 40%–60% of vaccinated allogeneic HCT recipients within 12 months after HCT and that immunogenicity would be lower in patients vaccinated earlier. At the study design stage, a power calculation was performed for detecting a difference in immunogenicity rates between cohorts with early and late vaccination relative to HCT; unequal enrollment to these cohorts was expected, with 118 and 43 projected to be allocated to early and late cohorts, respectively. It was determined that at least 81% power would be provided to detect a difference of 25% in immunogenicity rates between cohorts (Supplemental Methods; Table S16).

Anti-S IgG, neutralizing antibodies, and TCR results are displayed in box-and-whisker plots and compared using nonparametric Wilcoxon rank sum tests that are robust to features such as skewness and high dispersion which may arise in immunogenicity endpoints. To determine relevant anti-S IgG thresholds for immunogenicity, receiver operating characteristic (ROC) curves were employed using anti-S IgG as a continuous marker and dichotomous outcomes of neutralizing antibodies at any level (≥40 ID50), neutralizing antibodies at the median level (≥5274 ID50) achieved in a non-immunocompromised cohort vaccinated with two doses of mRNA-1273 (Moderna) in a clinical trial using the same assay,^32^ and qualitative SARS-CoV-2-specific T-cell responses. Anti-S IgG values corresponding to neutralizing antibodies ≥5274 ID50 were subsequently considered positive responses. The proportion of participants with an anti-S IgG positive response are described with Wald 99% confidence intervals (CIs); response rates were compared using a two-sided Z test of the difference in proportions between timing strata. To adjust for imbalances in baseline variables between timing cohorts, propensity scores for the likelihood of being in the <4-month cohort were constructed using logistic regression with stepwise variable selection. Variables from Tables 1 and 2 with p-values <0.05 were included in the model. A propensity-adjusted analysis compared positive anti-S IgG responses at the post-V2, post-V3, and end-of-study time points between the <4 month and 4–12-month cohorts using a Mantel–Haenszel test with strata determined by the quintiles of the propensity score distribution. Stratified estimates and 99% Wald CIs are presented for the odds ratio of response between timing cohorts. Logistic and linear regression models evaluated the impact of vaccination timing on anti-S IgG positive responses with adjustment for other covariates determined by bidirectional stepwise selection, with vaccine timing forced into the model and p-values <0.05 as the criterion for inclusion of covariates. All participants were included in analyses as relevant (e.g., participants who did not receive a second vaccine were not excluded). Analyses were performed using SAS Version 9.4 and R version 4.2. A p-value threshold of 0.01 was used to determine significance for all statistical comparisons to account for multiple comparisons.

**Table 1:** Demographic and clinical characteristics of the participants.

Characteristic	<4-month cohort	4–12-month cohort	p-value^d^	Overall
No. of patients	76	99		175
Median duration of follow up (months)	8.06 (7.07–8.72)	7.76 (7.11–8.29)	0.62	7.86 (7.07–8.39)
Age at HCT, years - no. (%)				
Median (range)	58.3 (9.3–75.4)	58.7 (10.4–76.7)	0.85	58.3 (9.3–76.7)
<18	4 (5)	4 (4)		8 (5)
18–29	6 (8)	8 (8)		14 (8)
30–39	2 (3)	11 (11)		13 (7)
40–49	11 (14)	13 (13)		24 (14)
50–59	20 (26)	17 (17)		37 (21)
60–69	27 (36)	35 (35)		62 (35)
≥70	6 (8)	11 (11)		17 (10)
Sex - no. (%)			1.00	
Female	35 (46)	46 (46)		81 (46)
Male	41 (54)	53 (54)		94 (54)
Hispanic or Latinx ethnicity- no. (%)			0.47	
Yes	2 (3)	6 (6)		8 (5)
No	72 (94)	92 (93)		163 (94)
Unknown/Not reported	2 (3)	1 (1)		3 (2)
Race or Ethnic Group other than Hispanic or Latinx - no. (%)			0.87	
White	61 (85)	78 (85)		139 (85)
Black	5 (7)	6 (7)		11 (7)
Asian	4 (6)	6 (7)		10 (6)
More than one race	2 (3)	1 (1)		3 (2)
Unknown/Not reported	2	2		4
Underlying Disease^a^ - no. (%)			0.21	
AML/MDS/MPN	54 (71)	64 (65)		118 (67)
ALL/other leukemia/aplastic anemia	12 (16)	26 (26)		38 (22)
Others	10 (13)	9 (9)		19 (11)
Date of HCT - no. (%)			<0.01	
May, 2020–December, 2020	0 (0)	22 (22)		22 (13)
January, 2021–March, 2021	15 (20)	35 (35)		50 (29)
April, 2021–June, 2021	40 (53)	35 (35)		75 (43)
July, 2021–August, 2021	21 (28)	7 (7)		28 (16)
Number of enrolling centers	16	21		25
Graft source - no. (%)			<0.01	
Bone marrow	4 (5)	16 (16)		20 (11)
Peripheral blood	72 (95)	79 (80)		151 (86)
Cord blood	0 (0)	4 (4)		4 (2)
Donor and HLA match - no. (%)			0.24	
Matched related	19 (25)	14 (14)		33 (19)
Matched unrelated	33 (43)	42 (42)		75 (43)
Mismatched related	17 (22)	31 (31)		48 (27)
Mismatched unrelated	7 (9)	12 (12)		19 (11)
Conditioning intensity - no. (%)			0.85	
Myeloablative	34 (45)	40 (40)		74 (42)
Reduced intensity	26 (34)	37 (37)		63 (36)
Non-myeloablative or none	16 (21)	22 (22)		38 (22)
GVHD prophylaxis^b^^,^^c^ - no. (%)			0.87	
Post-HCT cyclophosphamide regimen	29 (38)	42 (42)		71 (41)
Non-post-HCT cyclophosphamide regimen	45 (59)	55 (56)		100 (57)
Unknown/Not reported	2 (3)	2 (2)		4 (2)
Acute GVHD prior to baseline sample - no. (%)			0.08	
No	56 (74)	60 (61)		116 (66)
Yes	20 (26)	39 (39)		59 (34)
Chronic GVHD prior to baseline sample - no. (%)			<0.01	
No	74 (97)	79 (80)		153 (87)
Yes	2 (3)	20 (20)		22 (13)
Immunosuppressive medications in use^b^^,^^c^ - no. (%)				
Pre-V1	65 (86)	73 (74)	0.06	138 (79)
Post-V1	64 (84)	67 (68)	0.01	131 (75)
Post-V2	59 (78)	60 (61)	0.02	119 (68)
Post-V3	36 (47)	35 (35)	0.12	71 (41)
End-of-study	27 (36)	37 (37)	0.87	64 (37)
Absolute lymphocyte count (cells/mm^3^) at baseline, median (IQR)	600 (400–800)	700 (500–1300)	<0.01	700 (400–1100)
Absolute CD19+ B-cell count at baseline			0.67	
Number of participants tested	59	68		127
Median (IQR), cells/mm^3^	92 (38–169)	87 (53–198)		92 (42–183)
Absolute CD4+ T-cell count at baseline			<0.01	
Number of participants tested	59	68		127
Median (IQR), cells/mm^3^	56 (26–111)	110 (40–262)		78 (34–159)

Percentages may not total 100 because of rounding. HCT indicates hematopoietic cell transplant; IQR, interquartile range; AML, acute myelogenous leukemia; MDS, myelodysplastic syndrome; MPN, myeloproliferative neoplasm; ALL, acute lymphoblastic leukemia; HLA, human leukocyte antigen; GVHD, graft-versus-host disease.

a The underlying disease ‘Others’ category consisted of non-Hodgkin lymphoma, Hodgkin lymphoma, sickle cell disease, and congenital immunodeficiencies or inborn errors of metabolism.

b T-cell depleting agents were administered in 8 (11%) participants in the <4-month-cohort and 18 (18%) of participants in the 4-12-month cohort.

c See Table S1 for details.

d Calculated by a Fisher exact test or Kruskal–Wallis test as appropriate.

**Table 2:** SARS-CoV-2 vaccination, infection, and treatment characteristics of the participants.

Characteristic	<4-month cohort	4–12-month cohort	p-value^c^	Overall
No. of patients	76	99		175
Donor Vaccination Status Pre-donation - no. (%)			<0.01	
No	2 (3)	20 (20)		22 (13)
Yes	6 (8)	1 (1)		7 (4)
Unknown/Not reported	68 (89)	78 (79)		146 (83)
SARS-CoV-2 infection - no. (%)			0.82	
No infection	62 (82)	84 (85)		146 (83)
Prior to baseline	9 (12)	9 (9)	0.58	18 (10)
Before HCT	3 (4)	1 (1)		4 (2)
After HCT	0 (0)	1 (1)		1 (1)
Date Unknown	6 (8)	7 (7)		13 (7)
After initiating vaccination	5 (7)	6 (6)		11 (6)
Participant vaccinated prior to HCT - no. (%)			0.01	
No	58 (76)	90 (91)		148 (85)
Yes	18 (24)	9 (9)	1.00	27 (15)
Ad26.COV2.S	1 (1)	1 (1)		2 (1)
mRNA-1273	8 (11)	3 (3)		11 (6)
BNT16b2	8 (11)	4 (4)		12 (7)
Unknown/Not reported	1 (1)	1 (1)		2 (1)
Anti-Nucleocapsid IgG - no. (%)			0.45	
Negative	66 (87)	88 (89)		154 (88)
Positive	10 (13)	9 (9)		19 (11)
Unknown/Not reported	0 (0)	2 (2)		2 (1)
Received Vaccine dose 1 - no. (%)				
No	0 (0)	0 (0)		0 (0)
Yes	76 (100)	99 (100)	0.72	175 (100)
Ad26.COV2.S	1 (1)	0 (0)		1 (1)
mRNA-1273	21 (28)	28 (28)		49 (28)
BNT16b2	54 (71)	71 (72)		125 (71)
Time from HCT to vaccine dose 1, month - median (range)	3.4 (2.2–3.9)	5.8 (4.0–11.8)	<0.01	4.2 (2.2–11.8)
Received Vaccine dose 2 - no. (%)			1.00	
No	3 (4)	4 (4)		7 (4)
Yes	73 (96)	95 (96)	0.86	168 (96)
mRNA-1273	21 (28)	26 (26)		47 (27)
BNT16b2	52 (68)	69 (70)		121 (69)
Time from HCT to vaccine dose 2, month - median (range)	4.2 (2.9–5.1)	6.7 (4.7–13.4)	<0.01	5.0 (2.9–13.4)
Time between vaccine dose 1 and 2, month - median (range)	0.79 (0.5–1.79)	0.75 (0.54–9.86)	0.79	0.75 (0.5–9.86)
Received Vaccine dose 3 - no. (%)			0.47	
No	15 (20)	25 (24)		40 (23)
Yes	61 (80)	74 (76)	0.11	135 (77)
mRNA-1273	19 (25)	14 (14)		33 (19)
BNT16b2	42 (55)	60 (61)		102 (58)
Time from HCT to vaccine dose 3, month - median (range)	6.1 (4.9–11.6)	9.1 (5.8–17.9)	<0.01	7.6 (4.9–17.9)
Time between vaccine dose 2 and 3, month - median (range)	1.6 (0.9–7.4)	2.1 (0.9–6.8)	0.29	1.9 (0.9–7.4)
Received Vaccine dose 4 - no. (%)			1.00	
No	72 (95)	93 (94)		165 (94)
Yes	4 (5)	6 (6)	1.00	10 (6)
mRNA-1273	1 (1)	1 (1)		2 (1)
BNT16b2	3 (4)	5 (5)		8 (5)
Time from HCT to vaccine dose 4, month - median (range)	9.8 (9.1–10.2)	12.2 (8.8–14.0)	0.12	11.1 (8.8–14.0)
Time between vaccine dose 3 and 4, month - median (range)	4.1 (3.6–6.5)	4.7 (1.8–5.3)	0.91	4.4 (1.8–6.5)
Receipt of tixagevimab-cilgavimab (Evusheld)^a^ - no. (%)				
Pre-V1	0 (0)	1 (1)	1.00	1 (1)
Post-V1	0 (0)	0 (0)		0 (0)
Post-V2	3 (4)	2 (2)	0.65	5 (3)
Post-V3	2 (3)	2 (2)	1.00	4 (2)
End-of-study	18 (24)	16 (16)	0.25	34 (19)
Receipt of IVIG^b^ - no. (%)				
Pre-V1	7 (9)	2 (2)	0.04	9 (5)
Post-V1	2 (3)	3 (3)	1.00	5 (3)
Post-V2	1 (1)	3 (3)	0.63	4 (2)
Post-V3	2 (3)	3 (3)	1.00	5 (3)
End-of-study	4 (5)	4 (4)	0.73	8 (5)

Percentages may not total 100 because of rounding. HCT indicates hematopoietic cell transplant; IVIG, intravenous immunoglobulin.

a Within 6 months prior to sample collection.

b Within 1 month prior to sample collection.

c Calculated by a Fisher exact test or Kruskal–Wallis test as appropriate.

### Role of the funding source

The funders of the study had no role in study design, data collection, data analysis, data interpretation, or writing of the report. JAH, MJM, JK, MMH, MLR, and M-AP had access to the dataset. All study authors had final responsibility for the decision to submit for publication.

## Results

### Participants and treatment characteristics

We enrolled 175 allogeneic HCT recipients from 22 centers in the United States between April 22, 2021 and November 17, 2021; 76 (43%) participants were vaccinated <4 months after HCT and 99 (57%) 4–12 months after HCT. Demographic and baseline characteristics are summarized in Table 1 and were generally similar between cohorts. Most individuals were adults with acute leukemia. Acute and chronic GVHD were diagnosed prior to the first vaccine in 34% and 13% of participants, respectively. Most individuals (79%) were taking immunosuppressive medications at the time of the first vaccine (Table 1 and Table S1). Table 2 displays SARS-CoV-2 vaccination, infection, and treatment characteristics of study participants. BNT16b2 (Pfizer-BioNTech) was the most frequent mRNA SARS-CoV-2 vaccine, and only one individual received the Ad26.COV2.S vaccine (Johnson and Johnson). Prior SARS-CoV-2 infection and pre-HCT vaccination of the recipient and/or donor were more frequent in the <4-month cohort. Seven participants did not receive a second dose, 40 did not receive a third dose, and only 10 participants received four doses; distributions were similar between cohorts. The subgroup of 60 participants who were tested for neutralizing antibodies and SARS-CoV-2-specific TCRs was similar to the overall cohort (Table S2).

The median time to vaccination post-HCT was 3.4 months (interquartile range [IQR], 3.3–3.7) in the <4-month cohort and 5.8 months (IQR, 4.6–7.5) in the 4–12-month cohort (Fig. S2). Receipt of tixagevimab-cilgavimab (Evusheld) and immunoglobulin replacement therapy (IGRT) was infrequent but equally distributed between cohorts. Five (7%) participants in the <4-month cohort and six (6%) in the 4-12-month cohort were diagnosed with SARS-CoV-2 infections after initiating vaccinations (Table 2). No patients were treated with SARS-CoV-2-specific monoclonal antibodies.

### Binding and neutralizing antibodies

**Fig. 1:**
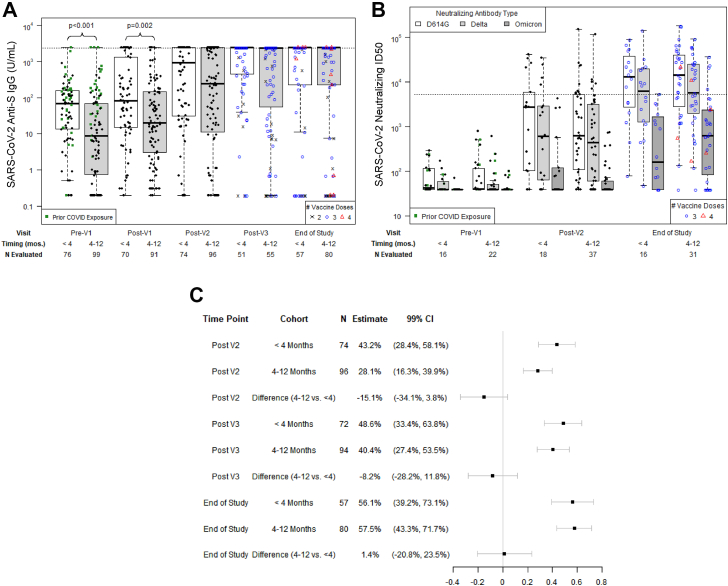
Longitudinal SARS-CoV-2 antibody responses. **A)** SARS-CoV-2 anti-S IgG titers per time point. The horizontal dotted line indicates the threshold for a positive response, defined as anti-S IgG ≥2403 U/mL as determined from a ROC curve analysis. The maximum reported value was 2500 U/mL (10^3.4^ U/mL). **B)** SARS-CoV-2 neutralizing titers in a subgroup of 60 participants; ID50 is defined as the reciprocal of the sample dilution required to reduce relative luminescence units by 50%. The horizontal dotted line shows the median neutralizing antibody level (5274 ID50) achieved in a healthy cohort vaccinated with two doses of mRNA-1273 (Moderna) in a clinical trial and tested with the same assay and defined here as a positive response. In both A and B, prior COVID exposure identifies participants with a known prior SARS-CoV-2 infection, prior SARS-Cov-2 vaccination in the participant or stem cell donor, or positive anti-N IgG assay at baseline. **C)** Forest plot of the proportion of individuals at each time point, stratified by vaccine initiation <4 months versus 4–12 months after allogeneic HCT, who had a positive anti-S response; Wald 99% confidence intervals (CI) are shown.

At baseline (pre-V1), anti-N IgG was positive in 19 individuals (Table 2). Median anti-S IgG titers were higher in the <4-month cohort at the pre-V1 and post-V1 time points but similar subsequently (Fig. 1A; Table S3). In both cohorts, anti-S IgG increased at post-V2 and post-V3 timepoints (Table S4). Median anti-S IgG was >2500 U/mL at the post-V3 and end-of-study time points. Neutralizing antibody titers were similar at pre-V1, post-V2, and end-of-study time points in the <4-month versus 4–12-month cohorts (Fig. 1B; Table S2). Neutralizing antibody titers increased for each SARS-CoV-2 strain over time (Table S3) and were highest for Wuhan D614G and lowest for Omicron B.1.1.529. Most participants had low neutralizing antibody levels for Omicron B.1.1.529 at the end-of-study time point.

An ROC curve analysis demonstrated that anti-S IgG ≥15.6 U/mL and ≥2403 U/mL had high sensitivity and specificity for detecting any and high-level neutralizing antibodies, respectively (Fig. S3A and S3B). A positive response, based on the threshold of anti-S IgG ≥2403 U/mL, was detected in a similar proportion of individuals in the <4-month and 4-12-month cohorts at post-V2, post-V3, and end-of-study time points (Fig. 1C). These findings were recapitulated in a propensity-adjusted analysis (Table S5). Propensity score models, as well as pre- and post-adjusted standardized mean deviations (SMDs), are depicted in Tables S6 and S7.

Among participants receiving IGRT within a month prior to a sample collection, there were similar anti-S IgG and neutralizing antibody titers compared to those not receiving IGRT (Tables S8 and S9). However, patients receiving prophylaxis with tixagevimab-cilgavimab within six months prior to a sample had higher anti-S IgG titers; comparisons for neutralizing antibody titers were limited by low numbers (Tables S10 and S11).

Anti-S IgG titers were similar when stratified by vaccine type (Fig. S4). Participants with documented SARS-CoV-2 infection after initiating vaccinations had higher subsequent anti-S IgG titers than uninfected individuals (Table S12), and none had preceding anti-S IgG titers ≥2403 U/mL.

### SARS-CoV-2-specific TCRs

**Fig. 2:**
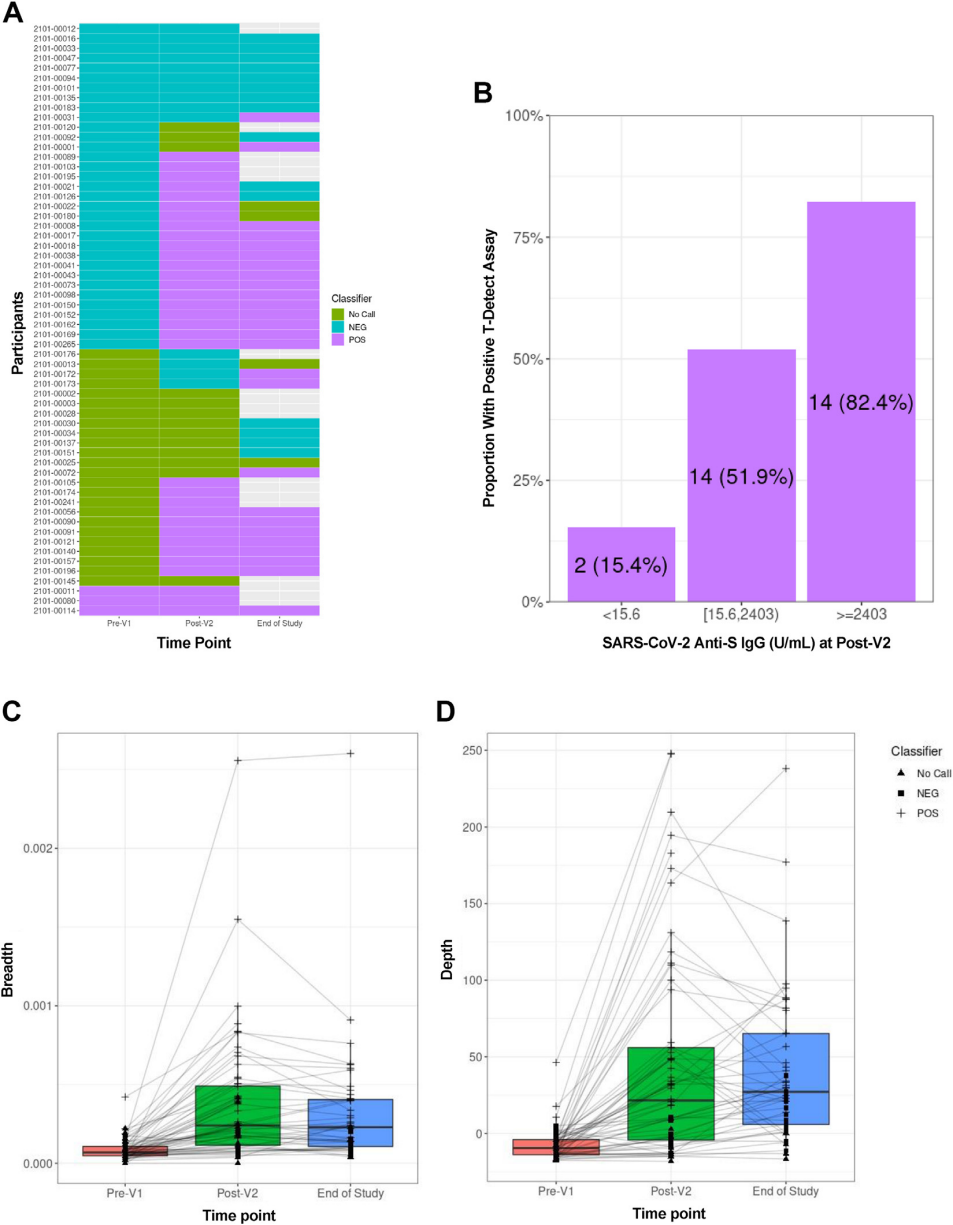
SARS-CoV-2-specific T-cell receptor (TCR) variable beta chain sequencing results in a subgroup of 60 participants vaccinated <4 months (n = 19) or 4–12 months (n = 41) after allogeneic HCT. **A)** Qualitative results indicating a positive, negative, or indeterminate result for the presence of SARS-CoV-2-specific TCRs based on the T-Detect ImmunoSEQ Assay classifier (Adaptive Biotechnologies, Seattle, WA). Each row indicates a unique participant. Unfilled cells at the end-of-study time point indicate that no sample was available for testing. **B)** The proportion of participants with a positive T-Detect at the post-V2 timepoint in categories of negative (n = 13), any detectable (n = 27), or positive (n = 17) SARS-CoV-2 anti-S IgG titers; three individuals with a positive T-Detect at the pre-V1 time point were excluded. (**C** and **D)** Quantitative values at each time point indicating the SARS-CoV-2 TCR breadth (**C**), defined as the proportion of total unique TCRs associated with SARS-CoV-2; and depth (**D**), defined as the extent to which SARS-CoV-2-associated TCRs expand.

At pre-V1, three participants had a positive T-Detect for SARS-CoV-2-specific T-cells (Fig. 2A). After excluding these individuals, 30 (53%) of the remaining 57 participants had a positive T-Detect at the post-V2 time point. Among 45 participants with samples tested at end-of-study, 26 (58%) had a positive T-Detect. When stratified by cohort, a similar proportion of participants had a positive T-Detect assay at the post-V2 time point in the <4-month (53.3%) and 4-12-month cohorts (52.4%). A positive T-Detect assay was observed in 2/13 (15.4%), 14/27 (51.9%), and 14/17 (82.4%) participants in the no, low, and positive SARS-CoV-2 anti-S IgG categories, respectively (Fig. 2B). An ROC curve analysis demonstrated that anti-S IgG ≥384 U/mL had moderate sensitivity (76%) and specificity (78%) for identifying individuals who also had a T-cell response (Fig. S3C).

We next quantified the relative number (breadth) and relative sum frequency (depth) of detectable SARS-CoV-2 TCRs. We observed increased depth and breadth at the post-V2 time point, with stable results at the end-of-study time point (Fig. 2C and D). When stratified by categories of SARS-CoV-2 anti-S IgG titers, both breadth and depth increased as the anti-S IgG titer increased (Fig. S5). Breadth and depth of SARS-CoV-2 TCRs were similar when stratified by vaccine type (Fig. S6).

### Predictors of SARS-CoV-2 mRNA vaccine immunogenicity

Adjusted regression models identified associations of prior SARS-CoV-2 infection and/or recipient vaccination with a positive response (anti-S IgG ≥2403 U/mL) at post-V2, higher CD19+ B- and CD4+ T-cell counts with a positive response at post-V2 only, and younger age with a positive response at the end-of-study (Tables S13 and S14). Vaccine initiation timing was not associated with a positive response when analyzed as a categorical (<4 versus 4–12 months or <6 versus 6–12 months; Table S13) or continuous variable (Table S14). Data are also depicted in scatter plots in Fig. S7. Notably, prior GVHD or use of immunosuppressive medications were not associated with a positive response.

### Safety of SARS-CoV-2 mRNA vaccines

Possible vaccine-related grade 3 or higher adverse events were uncommon (Table 3). New-onset acute or chronic GVHD were within the expected ranges for this patient population.

**Table 3:** New graft-versus-host disease (GVHD) events and grade 3 or higher adverse events possibly related to SARS-CoV-2 mRNA vaccination reported at each time point.

Event	<4-month cohort				4-12-month cohort			
	Post-V1 (n = 76)	Post-V2 (n = 73)	Post-V3 (n = 53)	End-of-study (n = 57)	Post-V1 (n = 96)	Post-V2 (n = 88)	Post-V3 (n = 65)	End-of-study (n = 76)
**New-onset GVHD**								
**Acute GVHD skin grade**								
1	1	–	–	–	1	1	3	1
2	1	3	–	2	1	–	–	–
3	–	–	1	–	1	1	–	–
**Acute GVHD upper GI**								
No	2	3	1	3	5	2	3	2
Yes	–	1	1	1	1	–	–	–
**Acute GVHD lower GI grade**								
1	–	1	1	–	1	–	–	–
3	–	–	–	–	1	–	–	–
4	–	1	–	–	–	–	–	–
**Acute GVHD liver grade**								
1	–	–	–	–	1	1	–	–
2	–	–	–	1	–	–	–	–
**Chronic GVHD severity**								
Mild	3	4	4	10	13	14	9	12
Moderate	1	6	5	6	5	8	4	3
Severe	–	–	–	–	–	–	–	–
**Chronic GVHD extent**								
Limited	4	4	4	10	16	14	7	10
Extensive	1	6	5	6	2	9	6	6
**Adverse events ≥ Grade 3**								
Fever	–	–	–	–	–	2	–	–
Fatigue	1	1	–	–	–	–	–	–
Allergic reaction	–	–	–	1	–	–	–	1
Nausea	–	–	–	2	–	1	–	–
Vomiting	–	–	–	2	–	–	–	–
Diarrhea	–	–	–	1	–	–	–	–
Cystitis noninfective	–	–	–	–	–	–	–	1
Acute kidney injury	–	–	–	1	–	–	–	–
Dialysis	–	–	–	–	–	1	–	–
Hypotension	–	–	–	–	–	–	–	1
Hypertension	–	1	–	–	2	1	–	–
Pericardial effusion	–	–	–	1	1	–	–	–
Thromboembolic event	–	1	–	–	–	–	–	–
Arthralgia (joint pain)	–	–	–	–	–	1	–	–
Myalgia (muscle pain)	–	1	–	–	–	1	–	1
Hypoxia	–	–	–	1	–	–	–	2
Dyspnea	–	–	–	1	–	–	1	–
Hyperglycemia	–	–	–	–	–	–	1	1
Hepatitis	2	2	–	–	4	1	–	–

Adverse events (according to NCI CTCAE Version 5.0) are shown for those documented prior to the indicated time point. Participants may have the same adverse event in multiple visits. GVHD body site involvement did not correlate with adverse events at that site (e.g., rash did not correlate with skin GVHD).

## Discussion

In this prospective study of allogeneic HCT recipients receiving mRNA SARS-CoV-2 vaccinations within the first 12 months after HCT, we demonstrate that humoral and cellular responses after two or more vaccinations were similar in participants initiating vaccination <4 months versus 4–12 months after HCT. We determined that anti-S IgG titers ≥2403 U/mL had high sensitivity and specificity for the presence of neutralizing titers similar to those observed in non-immunocompromised individuals, and 57% of participants had anti-S IgG ≥2403 U/mL at the final time point. This did not appear to be impacted by use of immunosuppressive medications or a diagnosis of GVHD. Additionally, most individuals achieving this anti-S IgG level also had SARS-CoV-2-specific T-cell responses. Together, these data support starting mRNA SARS-CoV-2 vaccination three months after HCT, irrespective of concurrent GVHD or use of immunosuppressive medications.

Based on historical data for other vaccines in allogeneic HCT recipients, we hypothesized that SARS-CoV-2 vaccines would be immunogenic in 40%–60% of patients vaccinated within 12 months after HCT, and that immunogenicity would be lower in patients vaccinated earlier. We observed immunogenicity rates within this range but no differences by timing of vaccine initiation post-HCT. Most guidelines recommend post-HCT vaccine initiation three to six months after HCT,^25^, ^26^, ^27^^,^^36^ whereas some suggest waiting six to twelve months,^29^ noting that these recommendations lack prospective validation. To the authors’ knowledge, this study is one of the largest prospective analyses of vaccination for any pathogen within the first year after allogeneic HCT and supports current guidelines for SARS-CoV-2 vaccination starting three months post-HCT.^30^^,^^31^ Additionally, there are few studies of mRNA vaccine formulations for other pathogens in HCT recipients. Our data provide encouraging proof-of-concept for the utility of early vaccination targeting additional pathogens with mRNA vaccine platforms.^37^

The observation that neutralizing antibody titers remained low at the end-of-study for the Omicron B.1.1.529 variant after Wuhan D614G-targeted vaccines underscores the importance of booster vaccinations, continued utilization and development of prophylactic and therapeutic interventions (e.g., monoclonal antibodies, virus-specific T-cell therapies, small molecule drugs), and other infection prevention strategies. Nonetheless, the detection of SARS-CoV-2-specific T-cell responses in 40% of participants with no or low anti-S IgG titers highlights the potential of vaccinations to mitigate disease severity in those who get infected.^24^^,^^38^ SARS-CoV-2-specific T-cell responses appear to peak after two vaccine doses, similar to findings in related contexts.^8^

In addition to the lack of association between time post-HCT and a positive anti-S IgG titer (≥2403 U/mL), we did not observe associations with other clinical factors often considered in heuristic approaches to vaccination timing, such as the presence of GVHD, use of immunosuppressive therapies, or absolute lymphocyte counts. Other studies have variably identified higher responses in patients receiving vaccines later post-HCT, as well as those with higher lymphocyte counts or without GVHD.^13^, ^14^, ^15^, ^16^^,^^18^, ^19^, ^20^^,^^22^

Strengths of this study include prospective, longitudinal sample and data collection in a representative cohort of allogeneic HCT recipients initiating SARS-CoV-2 mRNA vaccinations within the first 12 months after HCT, most of whom received three doses. We used neutralizing antibody results to determine a clinically meaningful anti-S IgG threshold for assessing humoral immunogenicity. We also evaluated cellular immunity with a novel immune repertoire profiling technique but note that it does not assess functional responses, how the results correlate with protection from severe disease is unknown, and some studies suggest that mRNA vaccines primarily induce CD4^+^ T-cell responses.^8^^,^^39^

A limitation is that this was an observational study, and we may not have fully accounted for confounding, although we used rigorous statistical methodology to account for observed differences. We are unable to fully account for variables may have affected vaccine initiation timing based on center policies or patient-specific clinical considerations. Because vaccine practices evolved over the course of the study, participants recruited earlier in the study were more likely to have initiated vaccination >4 months post-HCT and less likely to have had pre-HCT vaccination or receive cells from a vaccinated donor. This may explain the higher baseline and post-V1 anti-S titers observed in the <4-month cohort, although this difference was lost after second vaccinations. We also note that data were limited and/or unavailable for donors in regard to prior SARS-CoV-2 vaccination, prior infection, and SARS-CoV-2 anti-S IgG, which could impact recipient immunity.^40^ This study was not large enough to directly assess clinical efficacy. Due to the challenge of conducting studies with an endpoint of infection, antibody titers are accepted correlates for seroprotection in immunocompromised populations.^41^ We note that we did not account for the possibility of false positive or false negative antibody tests in the analyses, as these metrics are not established in this population. Only one individual received a non-mRNA vaccine, so these results only apply to SARS-CoV-2 mRNA vaccines, and a minority of participants received a fourth vaccine dose. There was limited enrollment of pediatric patients, as the study began before SARS-CoV-2 vaccines were approved for use in children. We note that subgroup comparisons were underpowered for definitive conclusions and are considered hypothesis-generating.

In conclusion, humoral and cellular responses were similar after two or more SARS-CoV-2 mRNA vaccinations in allogeneic HCT recipients vaccinated <4 months versus 4–12 months after HCT. Initiating the SARS-CoV-2 mRNA vaccination series between three to four months after allogeneic HCT should be routinely performed as an important component of a broader infection prevention strategy.

## Contributors

JAH, MLR, M-AP, MMH, and MJM designed the study; JAH, MLR, M-AP, MMH, MJM, LMG, JHY, and JA interpreted the results; MJM, MC, LWL, JK, SDW, JAH, JHY, and MLR analyzed the data and created the figures; JAH, JHY, KB, AB, RN, KP, AS, PA, PW, JM, CB, JM, MH, SDW, M-AP collected data; JAH, JHY, MLR drafted the manuscript. JAH, MJM, and JK accessed and verified the underlying data. All authors contributed to the writing and revision of the manuscript and approved the final version.

## Data sharing statement

The datasets generated and analyzed for this study are available from the corresponding author after publication upon reasonable request, without investigator support and with appropriate documentation of IRB approval and/or data access agreements as applicable.

## Declaration of interests

**J.A.H:** Research funding: *AlloVir;* Consulting: *Pfizer, Gilead, Moderna.*

**J-A.H.Y.:** Research funding: *AlloVir*.

**M.V.D.:** Research funding: *Janssen, Roche/Genentech*.

**S.D.W.:** Research funding: MSK Leukemia SPORE Career Enhancement Program and MSK Gerstner Physician Scholar program.

**M.H.:** Research Support/Funding: Takeda Pharmaceutical Company; ADC Therapeutics; Spectrum Pharmaceuticals; Astellas Pharma. Consultancy: Incyte Corporation, MorphoSys, SeaGen, Gamida Cell, Novartis, Legend Biotech, Kadmon, ADC Therapeutics; Omeros, Abbvie, Caribou, CRISPR, Genmab, Kite. Speaker's Bureau: Sanofi Genzyme, AstraZeneca, BeiGene, ADC Therapeutics, Kite. DMC: Myeloid Therapeutics, Inc.

**M.L.R.:** Research funding from Jazz Pharmaceuticals and Atara Bio-Pharma as well as employment by IQVIA Biotech.

**M-A.P.**: Honoraria from Adicet, AlloVir, Caribou Biosciences, Celgene, Bristol-Myers Squibb, Equilium, Exevir, Incyte, Karyopharm, Kite/Gilead, Merck, Miltenyi Biotec, MorphoSys, Nektar Therapeutics, Novartis, Omeros, OrcaBio, Syncopation, VectivBio AG, and Vor Biopharma. He serves on DSMBs for Cidara Therapeutics, Medigene, and Sellas Life Sciences, and the scientific advisory board of NexImmune. He has ownership interests in NexImmune and Omeros. He has received institutional research support for clinical trials from Incyte, Kite/Gilead, Miltenyi Biotec, Nektar Therapeutics, and Novartis.

All other authors report no relevant conflicts of interest.
