# Supplementary material for: SARS-CoV-2 vaccination in the first year after allogeneic hematopoietic cell transplant: a prospective, multicentre, observational study
Source: eClinicalMedicine. 2023 Apr 27;59:101983. doi: 10.1016/j.eclinm.2023.101983 (PMC10133891; doi:10.1016/j.eclinm.2023.101983)
Supplement: Supplementary material [file mmc1.docx]

**SUPPLEMENTARY APPENDIX TO**

**SARS-CoV-2 vaccination in the first year after allogeneic hematopoietic cell transplant: a prospective, multicentre, observational study**

[LIST OF INVESTIGATORS 3](#_Toc129696211)

[METHODS 4](#_Toc129696212)

[SUPPLEMENTARY FIGURES 7](#_Toc129696213)

[Figure S1. Enrollment and blood collection schema. 7](#_Toc129696214)

[Figure S2. Histogram of the month of first SARS-CoV-2 vaccination after allogeneic HCT. 8](#_Toc129696215)

[Figure S3. Receiver operating characteristic (ROC) curves of SARS-CoV-2 anti-S IgG titers as a predictor for neutralizing antibodies and a positive T-Detect for SARS-CoV-2-specific T-cells at the post-V2 time point. 9](#_Toc129696216)

[Figure S4. SARS-CoV-2 anti-S IgG titers stratified by vaccine type. 11](#_Toc129696217)

[Figure S5. SARS-CoV-2-specific T-cell receptor (TCR) variable beta chain sequencing results in a subgroup of 60 participants vaccinated either <4 months (n=19) or 4-12 months (n=41) after allogeneic HCT. 12](#_Toc129696218)

[Figure S6. SARS-CoV-2 anti-S IgG titers stratified by vaccine type. 13](#_Toc129696219)

[Figure S7. Scatter plots of SARS-CoV-2 anti-S IgG titers at the post-V2, post-V3, and end-of-study time points based on timing of initial vaccine initiation. 14](#_Toc129696220)

[SUPPLEMENTARY TABLES 15](#_Toc129696221)

[Table S1. GVHD regimens and pre-vaccine dose 1 immunosuppressive medications in use. 15](#_Toc129696222)

[Table S2. Demographic and clinical characteristics of the subgroup of participants tested for neutralizing antibodies and T-cell receptors^a^ for SARS-CoV-2. 16](#_Toc129696223)

[Table S3. Comparisons of anti-S IgG and neutralizing antibodies between the <4 month and 4-12 month vaccination timing cohorts by time point. 18](#_Toc129696224)

[Table S4. Comparisons of anti-S IgG and neutralizing antibodies at subsequent time points within each vaccination timing cohort (<4 month and 4-12 month). 18](#_Toc129696225)

[Table S5. Propensity score-stratified comparisons of SARS-CoV-2 anti-spike IgG positive response rates for participants initiating vaccinations <4 months versus 4-12 months after HCT, as well as <6 months versus 6-12 months. 19](#_Toc129696226)

[Table S6. Propensity score models of likelihood to be included in early vaccination timing cohorts. 20](#_Toc129696227)

[Table S7. Standardized mean differences of variables included in propensity score (PS) model for the likelihood of being in the 4-12 month vaccine timing cohort. 21](#_Toc129696228)

[Table S8. SARS-CoV-2 anti-S IgG titers among participants receiving immunoglobulin replacement therapy (IGRT) within a month prior to a sample collection. 22](#_Toc129696229)

[Table S9. SARS-CoV-2 neutralizing antibody titers among participants receiving immunoglobulin replacement therapy within a month prior to a sample collection. 23](#_Toc129696230)

[Table S10. SARS-CoV-2 anti-S IgG titers among participants receiving prophylaxis with tixagevimab-cilgavimab within 6 months of sample collection. 24](#_Toc129696231)

[Table S11. SARS-CoV-2 neutralizing antibody titers among participants receiving prophylaxis with tixagevimab-cilgavimab within 6 months of sample collection. 25](#_Toc129696232)

[Table S12. SARS-CoV-2 anti-S IgG titers among participants with subsequent SARS-CoV-2 infection. 26](#_Toc129696233)

[Table S13. Adjusted logistic regression models of the association of baseline and time-dependent variables with an observed positive SARS-CoV-2 anti-S IgG titer, *using vaccine timing as a categorical variable.* 27](#_Toc129696234)

[Table S14. Adjusted logistic regression models of the association of baseline and time-dependent variables with an observed positive SARS-CoV-2 anti-S IgG titer, *using vaccine timing as a continuous variable.* 29](#_Toc129696235)

[Table S15. Antibodies used for flow cytometry. 31](#_Toc129696236)

[Table S16: Power to Detect a 25% Difference in Immunogenicity Rates between 6-12 and <6 Month Cohorts with 41 and 112 Evaluable Patients (*a priori*). 32](#_Toc129696237)

[Table S17: Power to Detect a 25% Difference in Immunogenicity Rates between 4-12 and <4 Month Cohorts with 96 and 74 Evaluable Patients (*post-hoc*). 32](#_Toc129696238)

[REFERENCES 33](#_Toc129696239)

# LIST OF INVESTIGATORS

Paul Armistead, University of North Carolina Medical Center, Chapel Hill, NC; Jo-Anne Young, University of Minnesota, Minneapolis, MN; Jan Cerny, UMass Memorial Medical Center, Worcester, MA; Edward Copelan, Levine Cancer Institute, Charlotte, NC; David Epstein, Stanford Hospital, Stanford, CA; Aaron Etra, Mount Sinai Hospital, New York, NY; Mehdi Hamadani, Medical College of Wisconsin, Milwaukee, WI; Nancy Hardy, University of Maryland, College Park, MD; Joshua Hill, Fred Hutchinson Cancer Research Center, Seattle, WA; Kent Holland, Northside Hospital, Atlanta, GA; Mitch Horwitz, Duke University Hospital, Durham, NC; Dianna Howard, Wake Forest Baptist, Winston-Salem, NC; Robert Krance, Baylor College of Medicine, Houston, TX; Richard Maziarz, Oregon Health & Science University, Portland, OR; Philip McCarthy, Roswell Park Comprehensive Cancer Center, Buffalo, NY; John McCarty, Virginia Commonwealth University, Richmond, VA; Joseph McGuirk, University of Kansas, Lawrence, KS; Miguel Perales, Memorial Sloan Kettering Cancer Center, New York, NY; Ron Sobecks, Cleveland Clinic, Cleveland, OH; Lauren Veltri, West Virginia University, Morgantown, WV; Ned Waller, Emory University Hospital, Atlanta, GA; Peter Westervelt, Barnes-Jewish Hospital, Washington University, St. Louis, MO.

# METHODS

**Data Source and Collection**

The Center for International Blood and Marrow Transplant Research (CIBMTR) is a research consortium consisting of over 500 transplant centers internationally. Through a collaboration between the Medical College of Wisconsin and the National Marrow Donor Program, patient and outcomes data from these centers are collected and analyzed. Central auditing of the data is performed to ensure consistency and quality. The CIBMTR collects comprehensive demographic and clinical data prior to transplantation, at 100 days (D100), 6 months (D180), and 1 year after transplantation and annually thereafter. All patients included in this study gave written consent to participate in the CIBMTR Research Database and to have their data included in observational research. Participants also signed a study-specific consent for additional data and blood sample collection specific to SARS-CoV-2 vaccination and follow up as detailed in the manuscript. This study was approved by the institutional review boards of the Medical College of Wisconsin and the National Marrow Donor Program.

Data collected at baseline and each time point included demographics and HCT characteristics, medications (including for prevention or treatment of COVID-19), vaccine type, possible vaccine-related grade ≥3 adverse events according to NCI CTCAE Version 5.0, prior or new graft-versus-host disease (GVHD) and severity, and incident SARS-CoV2 infection.

The study was conducted under two CIBMTR repository protocols (“Protocol for a Research Database for Hematopoietic Cell Transplantation, Other Cellular Therapies and Marrow Toxic Injuries” and “Protocol for a Research Sample Repository for Hematopoietic Cell Transplantation, Other Cellular Therapies and Marrow Toxic Injuries”) that centers had ongoing institutional review board (IRB) approval for. The study-specific informed consent and assent for each center was approved by the National Marrow Donor Program (NMDP) single IRB.

**Participants and Study Design**

Prior SARS-CoV-2 infection, pre-hematopoietic SARS-CoV-2 vaccination, receipt of immunoglobulin replacement therapy, and receipt of prophylactic tixagevimab-cilgavimab were not exclusionary.

**Testing**

***Binding and neutralizing antibodies***. Total SARS-CoV-2 anti-spike receptor binding domain IgG was tested for in serum using the Roche Elecsys Anti-SARS-CoV-2 S electrochemiluminescence immunoassay (ECLIA).

Neutralizing antibody activity was measured in a FDA approved assay as previously described.^1^ Briefly, the assay uses lentiviral particles pseudotyped with full-length SARS-CoV-2 Spike protein and containing a firefly luciferase (Luc) reporter gene for quantitative measurements of infection by relative luminescence units (RLU). The backbone vector used in pseudovirus creation, F-lucP.CNDO∆U3, encodes the HIV genome with firefly luciferase replacing the HIV *env* gene. A codon-optimized version of the full-length spike gene of the Wuhan-1 SARS-CoV-2 strain (MN908947.3) (GenScript) was cloned into the Monogram proprietary *env* expression vector, pCXAS-PXMX. The D614G spike mutation was introduced into the original Wuhan sequence by site-directed mutagenesis. Pseudovirus infectivity was screened at multiple dilutions using HEK293 cells transiently transfected with ACE2 and TMPRSS2 expression vectors. RLUs were adjusted to ~ 50,000 for use in the neutralization assay. Neutralization was performed in 96-well plates by incubating pseudovirus with 10 serial three-fold dilutions of serum samples for one hour at 37 °C. Serum samples were heat-inactivated for 60 min at 56 °C prior to assay. The dilution series was based on a 1:20 starting dilution which was reported as 1:40 after addition of virus. Neutralization titers represent the inhibitory dilution (ID) of serum samples at which RLUs were reduced by 50% (ID50) compared to virus control wells (no serum wells). The units are 1/dilution, so an ID50 of 134 is a 1:134 dilution of the test serum at the 50% inhibition point.

Time points at which anti-S IgG was not detected (<0.4 U/mL) were not tested for neutralizing antibodies.

***Multiparametric Flow Cytometric Analysis*.** Cryopreserved peripheral blood mononuclear cells (PBMCs) were thawed, washed and resuspended in PBS, then incubated with Human TruStain FcX Fc receptor blocking solution (Biolegend) and Live/DEAD Fixable Blue Dead Cell Stain (Invitrogen) according to the manufacturers’ specifications for 20 minutes at room temperature (RT), protected from light. The cells were washed once in RPMI 1640 no phenol red + 4% FBS +0.01% sodium-azide and incubated with the antibody mix for 20 minutes at RT in the dark in the presence of Brilliant Staining Buffer (BD). The cells were washed, resuspended in 0.5% paraformaldehyde/PBS, and immediately acquired using a Cytek Aurora 5L flow cytometer (Cytek). The optimal concentration of all antibodies used in the study was defined by titration. Further information about the antibodies can be found in **Table S15**. For analysis, single-cell data was clustered using the FlowSOM R package and labeled using the Ek'Balam algorithm.^2,3^ Cell subset definitions were used as previously described.^4,5^ Cluster labeling, method implementation, and visualization were done through the Astrolabe Cytometry Platform (Astrolabe Diagnostics, Inc.).

**Statistical Analysis**

Antibody results from samples collected within six months of receipt of SARS-CoV-2-specific monoclonal antibodies were excluded and separately described.

We computed an *a priori* power calculation of the proportion of participants with immunogenicity between patients vaccinated 6-12 months versus <6 months after allogeneic HCT using a two sample Z test of the difference in proportions at a significance level of 5%. Immunogenicity for the a priori power calculation at the time of protocol development was defined as a ≥4-fold rise in anti-S IgG. The study was designed to provide at least 81% power to detect a 25% difference in immunogenicity response rates between timing cohorts by enrolling at least 118 and 43 patients to the <6 month and ≥6-12 month cohorts, respectively, assuming a 5% dropout rate (**Table S16**). Unequal allocation to the cohorts was expected based on the numbers of patients anticipated to enroll in these cohorts given clinical practice patterns at the time.

We also computed a *post-hoc* power calculation based on the sample sizes actually observed in the < 4 month and 4-12 month timing cohorts (**Table S17**). A power level of 90+% was attained to detect a 25% difference in immunogenicity rates based on the observed cohort sizes.

# SUPPLEMENTARY FIGURES

**
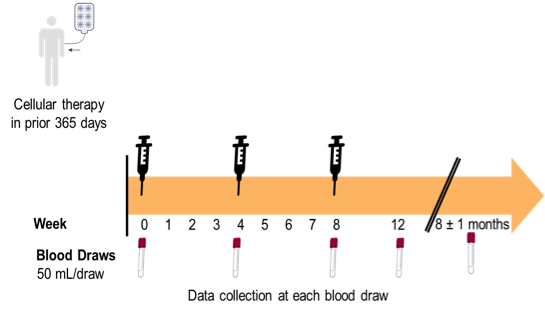
**

## Figure S1. Enrollment and blood collection schema.

## Figure S2. Histogram of the month of first SARS-CoV-2 vaccination after allogeneic HCT.

## Figure S3. Receiver operating characteristic (ROC) curves of SARS-CoV-2 anti-S IgG titers as a predictor for neutralizing antibodies and a positive T-Detect for SARS-CoV-2-specific T-cells at the post-V2 time point.

**A)** Estimated sensitivity and specificity of SARS-CoV-2 anti-S IgG for predicting detection of any neutralizing antibodies (threshold ≥40 ID50). **B)** Estimated sensitivity and specificity of SARS-CoV-2 anti-S IgG for predicting detection of neutralizing antibodies at the median level (5,274 ID50) achieved in a healthy cohort vaccinated with two doses of mRNA-1273 (Moderna) in a clinical trial and tested with the same assay.^30^ **C**) Estimated sensitivity and specificity of SARS-CoV-2 anti-S IgG for predicting a positive T-Detect result for SARS-CoV-2-specific T-cells. Patients who received tixagevimab-cilgavimab (Evusheld) within the prior six months were excluded for all analyses.

CI indicates confidence interval; AUC, area under the curve.

## Figure S4. SARS-CoV-2 anti-S IgG titers stratified by vaccine type.

There were no significant differences between anti-S IgG titers in participants receiving mRNA-1273 (Moderna) versus BNT16b2 (Pfizer-BioNTech) vaccines except at the post-V2 time point (p=0.033, Wilcoxon rank sum test comparing median levels). Participants who received a fourth vaccine dose were excluded from the end-of-study visit.

M indicates mRNA-1273 (Moderna); P, BNT16b2 (Pfizer-BioNTech); H, heterologous vaccination.

## Figure S5. SARS-CoV-2-specific T-cell receptor (TCR) variable beta chain sequencing results in a subgroup of 60 participants vaccinated either <4 months (n=19) or 4-12 months (n=41) after allogeneic HCT.

Quantitative values of SARS-CoV-2 TCR breadth (**A**) and depth (**B**) at the post-V2 time point in categories of negative, any detectable, or positive SARS-CoV-2 anti-S IgG titers.

## Figure S6. SARS-CoV-2 anti-S IgG titers stratified by vaccine type.

**A)** Post-V2 time point. **B)** End-of-study time point.

**
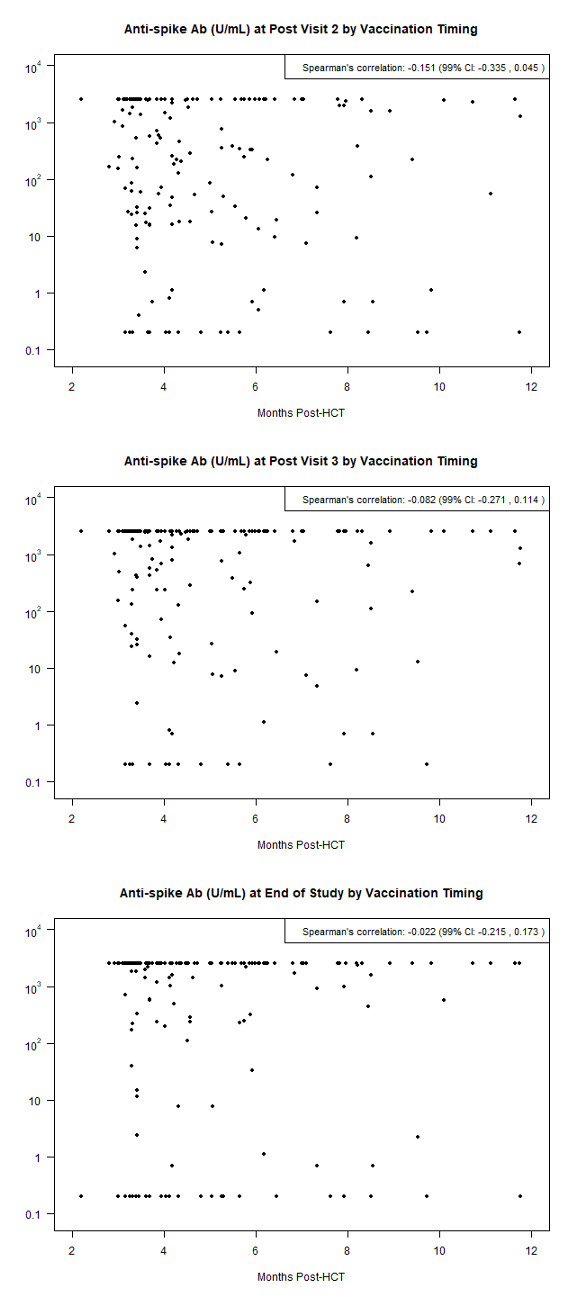
**

## Figure S7. Scatter plots of SARS-CoV-2 anti-S IgG titers at the post-V2, post-V3, and end-of-study time points based on timing of initial vaccine initiation.

# SUPPLEMENTARY TABLES

| Table S1. GVHD regimens and pre-vaccine dose 1 immunosuppressive medications in use. | | | |
| --- | --- | --- | --- |
| **Characteristic** | **<4-month cohort** | **4–12-month cohort** | **Overall** |
| No. of patients | 76 | 99 | 175 |
|  |  |  |  |
| Initial GVHD prophylaxis regimen - no. (%) |  |  |  |
| PtCy +/- others | 29 (38) | 42 (42) | 71 (41) |
| Non-PtCy regimens | 45 (59) | 55 (56) | 100 (57) |
| TCD +/- others | 1 (1) | 0 (0) | 1 (1) |
| CD34 selection +/- others | 0 (0) | 1 (1) | 1 (1) |
| CNI and MMF +/- others | 3 (4) | 14 (14) | 17 (10) |
| CNI and MTX +/- others | 34 (45) | 38 (38) | 72 (41) |
| CNI +/- others | 5 (7) | 1 (1) | 6 (3) |
| MTX +/- others | 2 (3) | 1 (1) | 3 (2) |
| Unknown/Not reported | 2 (3) | 2 (2) | 4 (2) |
|  |  |  |  |
| Pre-V1 immunosuppressive medications - no. (%) |  |  |  |
| CNI +/- other | 49 (64) | 40 (40) | 89 (51) |
| CNI and corticosteroids +/- other | 10 (13) | 17 (17) | 27 (15) |
| Corticosteroids +/- other (not CNI) | 2 (3) | 5 (5) | 7 (4) |
| TKI | 1 (1) | 6 (6) | 7 (4) |
| Cytotoxic therapy +/- other | 1 (1) | 2 (2) | 3 (2) |
| mTor inhibitor +/- other | 2 (3) | 2 (2) | 4 (2) |
| JAK-2 inhibitor | 0 (0) | 1 (1) | 1 (1) |
| None | 11 (14) | 26 (26) | 37 (21) |
| PtCy indicates post-hematopoietic transplant cyclophosphamide; TCD, T-cell depletion; CNI, calcineurin inhibitor; MMF, mycophenolate mofetil; MTX, methotrexate; TKI, tyrosine kinase inhibitor. | | | |

|  | Table S2. Demographic and clinical characteristics of the subgroup of participants tested for neutralizing antibodies and T-cell receptors^a^ for SARS-CoV-2. | | | | |
| --- | --- | --- | --- | --- | --- |
| **Characteristic** | | **<4 month cohort** | **4-12 month cohort** | **p-value^c^** | **Overall** |
| No. of patients | | 19 | 41 |  | 60 |
| Age at HCT, years - no. (%) | |  |  |  |  |
| Median (range) | | 52.1 (25.4-69.1) | 47.7 (10.4-72.2) | 0.87 | 48.2 (10.4-72.2) |
| <18 | | 0 (0) | 3 (7) |  | 3 (5) |
| 18-29 | | 3 (16) | 4 (10) |  | 7 (12) |
| 30-39 | | 1 (5) | 7 (17) |  | 8 (13) |
| 40-49 | | 5 (26) | 8 (20) |  | 13 (22) |
| 50-59 | | 3 (16) | 7 (17) |  | 10 (17) |
| 60-69 | | 7 (37) | 8 (20) |  | 15 (25) |
| ≥70 | | 0 (0) | 4 (10) |  | 4 (7) |
| Sex - no. (%) | |  |  | 0.78 |  |
| Female | | 9 (47) | 22 (54) |  | 31 (52) |
| Male | | 10 (53) | 19 (46) |  | 29 (48) |
| Hispanic or Latinx ethnicity- no. (%) | |  |  | 0.16 |  |
| Yes  Unknown/Not reported | | 0 (0)  2 (11) | 4 (10)  1 (2) |  | 4 (7)  3 (5) |
| Race or Ethnic Group other than Hispanic or Latinx - no. (%) | | | | 0.64 |  |
| White | | 13 (76) | 29 (71) |  | 42 (70) |
| Black | | 3 (18) | 6 (15) |  | 9 (15) |
| Asian | | 1 (6) | 4 (10) |  | 5 (9) |
| Unknown/Not reported | | 2 (11) | 2 (5) |  | 4 (10) |
| Underlying Disease - no. (%) | |  |  | 0.25 |  |
| AML/MDS/MPN | | 12 (63) | 24 (59) |  | 36 (60) |
| ALL/other leukemia/aplastic anemia | | 3 (16) | 13 (32) |  | 16 (27) |
| Others | | 4 (21) | 4 (10) |  | 8 (13) |
| Date of HCT - no. (%) | |  |  | <0.01 |  |
| May, 2020 – December, 2020 | | 0 (0) | 16 (39) |  | 16 (27) |
| January, 2021 – March, 2021 | | 13 (68) | 18 (44) |  | 31 (52) |
| April, 2021 – June, 2021 | | 6 (32) | 7 (17) |  | 13 (22) |
| Number of enrolling centers | | 8 | 14 |  | 16 |
| Graft source - no. (%) | |  |  | 0.06 |  |
| Bone marrow | | 0 (0) | 6 (15) |  | 6 (10) |
| Peripheral blood | | 19 (100) | 31 (76) |  | 50 (83) |
| Cord blood | | 0 (0) | 4 (10) |  | 4 (7) |
| Donor and HLA match - no. (%) | |  |  | 0.18 |  |
| Matched related | | 6 (32) | 8 (20) |  | 14 (23) |
| Matched unrelated | | 8 (42) | 12 (29) |  | 20 (33) |
| Mismatched related | | 5 (26) | 14 (34) |  | 19 (32) |
| Mismatched unrelated | | 0 (0) | 7 (17) |  | 7 (12) |
| Conditioning intensity - no. (%) | |  |  | 0.93 |  |
| Myeloablative | | 10 (56) | 19 (51) |  | 29 (53) |
| Reduced intensity | | 5 (28) | 11 (30) |  | 16 (29) |
| Non-myeloablative or none | | 4 (21) | 10 (24) |  | 14 (23) |
| GVHD prophylaxis^b^ - no. (%) | |  |  | 0.38 |  |
| Post-HCT cyclophosphamide regimen | | 5 (26) | 16 (39) |  | 21 (35) |
| Non-post-HCT cyclophosphamide regimen | | 14 (74) | 23 (56) |  | 37 (62) |
| Unknown/Not reported | | 0 (0) | 2 (5) |  | 2 (3) |
| Acute GVHD prior to baseline sample - no. (%) | |  |  | 0.05 |  |
| No | | 15 (79) | 21 (51) |  | 36 (60) |
| Yes | | 4 (21) | 20 (49) |  | 24 (40) |
| Chronic GVHD prior to baseline sample - no. (%) | |  |  | 0.01 |  |
| No | | 19 (58) | 30 (73) |  | 49 (82) |
| Yes | | 0 (42) | 11 (27) |  | 11 (18) |
| Immunosuppressive medications in use- no. (%) | |  |  |  |  |
| Pre-V1  Post-V1  Post-V2  Post-V3  End-of-study | | 17 (89)  17 (89)  15 (79)  11 (58)  7 (37) | 30 (73)  28 (68)  25 (61)  16 (39)  19 (46) | 0.19  0.11  0.24  0.26  0.58 | 47 (78)  45 (75)  40 (67)  27 (45)  26 (43) |
| Absolute lymphocyte count (cells/mm^3^) at baseline, median (IQR) | | 800 (600-900) | 1,200 (700-1,800) | 0.03 | 900 (600-1,700) |
| Absolute CD19+ B-cell count at baseline | |  |  | 0.97 |  |
| Number of participants tested | | 5 | 17 |  | 22 |
| Median (IQR), cells/mm^3^ | | 246 (159-359) | 315 (82-550) |  | 280 (82-550) |
| Absolute CD4+ T-cell count at baseline | |  |  | 0.22 |  |
| Number of participants tested | | 5 | 17 |  | 22 |
| Median (IQR), cells/mm^3^ | | 111 (63-137) | 267 (181-359) |  | 256 (111-359) |

Percentages may not total 100 because of rounding. HCT indicates hematopoietic cell transplant; IQR, interquartile range; AML, acute myelogenous leukemia; MDS, myelodysplastic syndrome; MPN, myeloproliferative neoplasm; ALL, acute lymphoblastic leukemia; HLA, human leukocyte antigen; GVHD, graft-versus-host disease.

^a^Three participants had a positive T-Detect for SARS-CoV-2-specific T-cells at baseline (all in the <4-month cohort) and were excluded from TCR analyses.

^b^T-cell depleting agents were administered in 0 participants in the <4-month-cohort and 5 (12%) of participants in the 4-12-month cohort.

^c^Calculated by a Fisher exact test or Kruskal-Wallis test as appropriate.

| Table S3. Comparisons of anti-S IgG and neutralizing antibodies between the <4 month and 4-12 month vaccination timing cohorts by time point, based on the data depicted in Figure 1A and 1B. | | | | | |
| --- | --- | --- | --- | --- | --- |
| **Test type** | **Time point** | | | | |
|  | **Pre-V1** | **Post-V1** | **Post-V2^a^** | **Post-V3** | **End-of-study^b^** |
| Anti-S IgG | <0.001 | 0.002 | 0.064 | 0.840 | 0.892 |
| Neutralizing, Wuhan D614G | 1.000 | NA | 0.654 | NA | 0.670 |
| Neutralizing, Delta B.1.617.2 | 0.807 | NA | 0.759 | NA | 0.553 |
| Neutralizing, Omicron B.1.1.529 | 0.268 | NA | 0.176 | NA | 0.369 |
| Wilcoxon rank sum test p-values are shown comparing median antibody levels between cohorts. Participants who received tixagevimab-cilgavimab (Evusheld) within 6 months prior to a visit were excluded. Participants who received a fourth vaccine dose (n=12) were excluded for the end-of-study time point.  ^a^For neutralizing antibodies, the comparison is between pre-V1 and post-V2.  ^b^For neutralizing antibodies, the comparison is between post-V2 and end-of-study. | | | | | |

| Table S4. Comparisons of anti-S IgG and neutralizing antibodies at subsequent time points within each vaccination timing cohort (<4 month and 4-12 month), based on the data depicted in Figure 1A and 1B. | | | | |
| --- | --- | --- | --- | --- |
| **Timing Cohort and test type** | **Time point** | | | |
|  | **Pre-V1 vs. Post-V1** | **Post-V1 vs. Post-V2^a^** | **Post-V2 vs. Post-V3** | **Post-V3 vs. end-of-study^b^** |
| **<4 months** |  |  |  |  |
| Anti-S IgG | 0.151 | <0.001 | <0.001 | 0.866 |
| Neutralizing, Wuhan D614G | NA | 0.006 | NA | 0.025 |
| Neutralizing, Delta B.1.617.2 | NA | 0.005 | NA | 0.016 |
| Neutralizing, Omicron B.1.1.529 | NA | 0.021 | NA | 0.147 |
| **4-12 months** |  |  |  |  |
| Anti-S IgG | 0.339 | <0.001 | <0.001 | 0.644 |
| Neutralizing, Wuhan D614G | NA | 0.001 | NA | <0.001 |
| Neutralizing, Delta B.1.617.2 | NA | 0.002 | NA | <0.001 |
| Neutralizing, Omicron B.1.1.529 | NA | 0.722 | NA | <0.001 |
| Wilcoxon signed rank test p-values are shown, testing whether median changes in antibody levels between visits differ from 0. Participants who received tixagevimab-cilgavimab (Evusheld) within 6 months prior to a visit were excluded. Participants who received a fourth vaccine dose (n=12) were excluded for the end-of-study time point.  ^a^For neutralizing antibodies, the comparison is between pre-V1 and post-V2.  ^b^For neutralizing antibodies, the comparison is between post-V2 and end-of-study. | | | | |

| Table S5. Propensity score-stratified comparisons of SARS-CoV-2 anti-spike IgG positive response rates for participants initiating vaccinations <4 months versus 4-12 months after HCT, as well as <6 months versus 6-12 months. | | | | |
| --- | --- | --- | --- | --- |
|  | **Odds ratio of response (4-12 months versus < 4 months)** | | |  |
| **Time point** | **Estimate** | **99% lower confidence limit** | **99% upper confidence limit** | **p-value** |
| Post-V2 | 0.542 | 0.212 | 1.388 | 0.096 |
| Post-V3 | 0.504 | 0.198 | 1.284 | 0.053 |
| End-of-study | 0.844 | 0.312 | 2.279 | 0.662 |
|  | **Odds ratio of response (6-12 months versus < 6 months)** | | |  |
| Post-V2 | 0.991 | 0.274 | 3.589 | 0.987 |
| Post-V3 | 0.839 | 0.243 | 2.899 | 0.720 |
| End-of-study | 1.035 | 0.297 | 3.606 | 0.943 |
| Response is defined as an anti-spike antibody level >2,403 U/mL. Propensity scores of being in the < 4-month timing cohort were constructed using stepwise variable selection; baseline absolute lymphocyte count and calendar date of HCT (May 2020-March 2021 vs. April 2021-June 2021 vs. July 2021-August 2021) were found to impact the likelihood of being in the <4 month cohort and were included in the model. Subsequently, a stratified analysis comparing response rates between timing cohorts was conducted in 5 strata determined by quintiles of the scores’ distribution. The same procedure was used to construct propensity scores for being in the < 6-month timing cohort, with baseline absolute lymphocyte count and calendar date of HCT also found to affect the chance of being in this cohort. Stratified odds ratio estimates and 95% Wald confidence intervals are provided. P-value is from a Mantel-Haenszel test. | | | | |

| Table S6. Propensity score models of likelihood to be included in early vaccination timing cohorts. | | | | | |
| --- | --- | --- | --- | --- | --- |
| **Propensity of being in 4-12 month timing cohort** | | | | | |
| **Variable^a^** | **Category** | **N** | **Odds Ratio** | **99% CI** | **p-value** |
| Calendar Date of HCT | May 2020 - March 2021 | 72 | 1.00 | - |  |
|  | April 2021 - June 2021 | 75 | 0.26 | (0.10, 0.69) | < 0.001 |
|  | July 2021 - August 2021 | 28 | 0.09 | (0.02, 0.37) | < 0.001 |
| Log10 Lymphocyte Count (10^6/L) |  | 175 | 2.93 | (0.78, 12.06) | 0.04 |
| **Propensity of being in 6-12 month timing cohort** | | | | | |
| Calendar Date of HCT | May 2020 - March 2021 | 72 | 1.00 | - |  |
|  | April 2021 - August 2021 | 103 | 0.04 | (0.01, 0.18) | < 0.001 |
| Log10 Lymphocyte Count (10^6/L) |  | 175 | 3.95 | (1.39, 15.87) | 0.002 |
| N indicates number; CI indicates confidence interval.  Logistic regression was used to construct propensity scores. A stepwise variable selection procedure was used to determine covariates to include in the models, with a p-value <0.05 as the criterion for inclusion. For each outcome, the displayed variables were the only variables retained in the adjusted model.  ^a^Covariates considered in models included age, sex, race, ethnicity, underlying disease, graft source, donor and HLA match, conditioning intensity, GVHD prophylaxis, anti-Nucleocapsid IgG, calendar date of HCT, treatment center, donor or recipient vaccination pre-enrollment, absolute lymphocyte count, absolute CD19+ B cell count, absolute CD4+ T cell count, receipt of Evusheld, receipt of IVIG, acute GVHD pre-enrollment, chronic GVHD pre-enrollment, immunosuppressive medication use, and recipient infection with SARS-CoV-2. | | | | | |

| Table S7. Standardized mean differences of variables included in propensity score (PS) model for the likelihood of being in the 4-12 month vaccine timing cohort. | | |
| --- | --- | --- |
| **Population^a^** | **Variable** | **Standardized mean difference**  **(4-12 mo. - <4 mo.)** |
| All (no PS adjustment) | Log lymphocyte count | -0.393 |
|  | HCT date of May 2020 - March 2021 | -0.843 |
|  | HCT date of April 2021 - June 2021 | 0.308 |
|  | HCT date of July 2021 - August 2021 | 0.425 |
| PS 1^st^ quartile | Log lymphocyte count | 0.018 |
|  | HCT date of May 2020 - March 2021 | -0.135 |
|  | HCT date of April 2021 - June 2021 | -0.067 |
|  | HCT date of July 2021 - August 2021 | 0.203 |
| PS 2^nd^ quartile | Log lymphocyte count | 0.076 |
|  | HCT date of May 2020 - March 2021 | 0.000 |
|  | HCT date of April 2021 - June 2021 | 0.042 |
|  | HCT date of July 2021 - August 2021 | -0.049 |
| PS 3^rd^ quartile | Log lymphocyte count | -0.050 |
|  | HCT date of May 2020 - March 2021 | 0.000 |
|  | HCT date of April 2021 - June 2021 | 0.000 |
|  | HCT date of July 2021 - August 2021 | 0.000 |
| PS 4^th^ quartile | Log lymphocyte count | 0.014 |
|  | HCT date of May 2020 - March 2021 | 0.000 |
|  | HCT date of April 2021 - June 2021 | 0.000 |
|  | HCT date of July 2021 - August 2021 | 0.000 |
| PS 5^th^ quartile | Log lymphocyte count | -0.017 |
|  | HCT date of May 2020 - March 2021 | -0.106 |
|  | HCT date of April 2021 - June 2021 | 0.084 |
|  | HCT date of July 2021 - August 2021 | 0.000 |
| Logistic regression was used to construct propensity scores. A stepwise variable selection procedure was used to determine covariates to include in the models, with a p-value <0.05 as the criterion for inclusion. For each outcome, the displayed variables were the only variables retained in the adjusted model. Patients were subsequently classified into 5 propensity score strata determined by quintiles of the scores’ distribution.  ^a^Standardized mean differences in variables are summarized for the entire study cohort (All) and separately within each propensity score stratum. | | |

| Table S8. SARS-CoV-2 anti-S IgG titers among participants receiving immunoglobulin replacement therapy (IGRT) within a month prior to a sample collection. | | | | | | | | |
| --- | --- | --- | --- | --- | --- | --- | --- | --- |
| **Time point** | **Received IVIG** | **N** | **Mean** | **Minimum** | **1st Quartile** | **Median** | **3rd Quartile** | **Maximum** |
| Pre-V1 | No | 158 | 156.89 | 0.20 | 2.90 | 27.65 | 119.00 | 2501.00 |
|  | Yes | 9 | 143.49 | 0.20 | 7.20 | 26.30 | 127.00 | 899.00 |
| Post-V1 | No | 142 | 494.24 | 0.30 | 7.70 | 43.45 | 336.00 | 2501.00 |
|  | Yes | 10 | 489.83 | 0.30 | 16.40 | 48.60 | 144.00 | 2501.00 |
| Post-V2 | No | 144 | 1174.68 | 0.20 | 28.70 | 560.00 | 2501.00 | 2501.00 |
|  | Yes | 10 | 1050.94 | 0.20 | 34.20 | 708.50 | 2420.00 | 2501.00 |
| Post-V3 | No | 98 | 1872.04 | 0.70 | 817.00 | 2501.00 | 2501.00 | 2501.00 |
|  | Yes | 6 | 2173.33 | 535.00 | 2501.00 | 2501.00 | 2501.00 | 2501.00 |
| End-of-study | No | 109 | 1826.69 | 0.20 | 994.00 | 2501.00 | 2501.00 | 2501.00 |
|  | Yes | 8 | 2188.46 | 0.70 | 2501.00 | 2501.00 | 2501.00 | 2501.00 |

| Table S9. SARS-CoV-2 neutralizing antibody titers among participants receiving immunoglobulin replacement therapy within a month prior to a sample collection. | | | | | | | | |
| --- | --- | --- | --- | --- | --- | --- | --- | --- |
| **Time point** | **Received IVIG** | **N** | **Mean** | **Minimum** | **1st Quartile** | **Median** | **3rd Quartile** | **Maximum** |
| **Wuhan D614G** | | | | | | | | |
| Pre-V1 | No | 34 | 112.77 | 39.90 | 39.90 | 42.08 | 118.08 | 797.63 |
|  | Yes | 3 | 39.90 | 39.90 | 39.90 | 39.90 | 39.90 | 39.90 |
| Post-V2 | No | 48 | 7463.77 | 39.90 | 98.32 | 641.59 | 4995.27 | 154204.0 |
|  | Yes | 3 | 997.45 | 565.37 | 565.37 | 857.16 | 1569.82 | 1569.82 |
| End-of-study | No | 43 | 27793.67 | 39.90 | 1851.61 | 11896.64 | 34901.34 | 182151.4 |
|  | Yes | 1 | 22626.57 | 22626.57 | 22626.57 | 22626.57 | 22626.57 | 22626.57 |
| **Delta B.1.617.2** | | | | | | | | |
| Pre-V1 | No | 34 | 74.52 | 39.90 | 39.90 | 39.90 | 53.29 | 614.70 |
|  | Yes | 3 | 39.90 | 39.90 | 39.90 | 39.90 | 39.90 | 39.90 |
| Post-V2 | No | 48 | 4704.06 | 39.90 | 54.68 | 405.95 | 2953.34 | 115451.0 |
|  | Yes | 3 | 365.97 | 230.98 | 230.98 | 311.40 | 555.53 | 555.53 |
| End-of-study | No | 43 | 14682.32 | 39.90 | 1262.13 | 5100.17 | 20635.42 | 93124.30 |
|  | Yes | 1 | 4675.75 | 4675.75 | 4675.75 | 4675.75 | 4675.75 | 4675.75 |
| **Omicron B.1.1.529** | | | | | | | | |
| Pre-V1 | No | 34 | 44.89 | 39.90 | 39.90 | 39.90 | 39.90 | 102.22 |
|  | Yes | 3 | 39.90 | 39.90 | 39.90 | 39.90 | 39.90 | 39.90 |
| Post-V2 | No | 48 | 221.28 | 39.90 | 39.90 | 39.90 | 63.88 | 4257.77 |
|  | Yes | 3 | 59.96 | 39.90 | 39.90 | 39.90 | 100.07 | 100.07 |
| End-of-study | No | 43 | 2956.81 | 39.90 | 39.90 | 598.50 | 2360.81 | 36897.97 |
|  | Yes | 1 | 119.71 | 119.71 | 119.71 | 119.71 | 119.71 | 119.71 |

| Table S10. SARS-CoV-2 anti-S IgG titers among participants receiving prophylaxis with tixagevimab-cilgavimab within 6 months of sample collection. | | | | | | | | |
| --- | --- | --- | --- | --- | --- | --- | --- | --- |
| **Time point** | **Received tixagevimab-cilgavimab** | **N** | **Mean** | **Minimum** | **1st Quartile** | **Median** | **3rd Quartile** | **Maximum** |
| Pre-V1 | No | 56 | 164.06 | 0.20 | 6.65 | 64.25 | 169.00 | 2501.00 |
|  | Yes | 3 | 1699.18 | 95.53 | 95.53 | 2501.00 | 2501.00 | 2501.00 |
| Post-V1 | No | 67 | 534.20 | 0.30 | 6.60 | 42.60 | 336.00 | 2501.00 |
|  | Yes | 4 | 1682.51 | 573.00 | 864.03 | 1828.03 | 2501.00 | 2501.00 |
| Post-V2 | No | 85 | 1071.76 | 0.20 | 24.00 | 331.00 | 2501.00 | 2501.00 |
|  | Yes | 8 | 1875.80 | 0.20 | 1250.60 | 2501.00 | 2501.00 | 2501.00 |
| Post-V3 | No | 59 | 1741.79 | 0.70 | 393.00 | 2501.00 | 2501.00 | 2501.00 |
|  | Yes | 13 | 2248.58 | 39.50 | 2501.00 | 2501.00 | 2501.00 | 2501.00 |
| End-of-study | No | 96 | 1572.18 | 0.20 | 140.50 | 2501.00 | 2501.00 | 2501.00 |
|  | Yes | 34 | 2047.02 | 0.20 | 1932.00 | 2501.00 | 2501.00 | 2501.00 |

| Table S11. SARS-CoV-2 neutralizing antibody titers among participants receiving prophylaxis with tixagevimab-cilgavimab within 6 months of sample collection. | | | | | | | | |
| --- | --- | --- | --- | --- | --- | --- | --- | --- |
| **Time point** | **Received tixagevimab-cilgavimab** | **N** | **Mean** | **Minimum** | **1st Quartile** | **Median** | **3rd Quartile** | **Maximum** |
| **Wuhan D614G** | | | | | | | | |
| Pre-V1 | No | 10 | 194.03 | 39.90 | 39.90 | 79.87 | 291.30 | 797.63 |
|  | Yes | 0 | - | - | - | - | - | - |
| Post-V2 | No | 30 | 7084.26 | 55.31 | 326.93 | 2471.39 | 5928.94 | 41500.61 |
|  | Yes | 0 | - | - | - | - | - | - |
| End-of-study | No | 32 | 31916.59 | 39.90 | 1981.69 | 12198.70 | 42874.25 | 182151.4 |
|  | Yes | 14 | 22511.37 | 462.84 | 3521.16 | 19856.24 | 34901.34 | 69092.68 |
| **Delta B.1.617.2** | | | | | | | | |
| Pre-V1 | No | 10 | 125.86 | 39.90 | 39.90 | 53.58 | 116.93 | 614.70 |
|  | Yes | 0 | - | - | - | - | - | - |
| Post-V2 | No | 30 | 4269.08 | 39.90 | 118.58 | 1090.99 | 3730.04 | 35054.11 |
|  | Yes | 0 | - | - | - | - | - | - |
| End-of-study | No | 32 | 14325.60 | 39.90 | 1433.01 | 5021.47 | 17877.71 | 93124.30 |
|  | Yes | 14 | 24159.03 | 458.20 | 1387.95 | 14285.24 | 25584.23 | 144935.2 |
| **Omicron B.1.1.529** | | | | | | | | |
| Pre-V1 | No | 10 | 47.29 | 39.90 | 39.90 | 39.90 | 39.90 | 81.60 |
|  | Yes | 0 | - | - | - | - | - | - |
| Post-V2 | No | 30 | 348.31 | 39.90 | 39.90 | 46.02 | 134.28 | 4257.77 |
|  | Yes | 0 | - | - | - | - | - | - |
| End-of-study | No | 32 | 3895.99 | 39.90 | 121.11 | 811.22 | 3322.61 | 36897.97 |
|  | Yes | 14 | 513.95 | 39.90 | 39.90 | 75.89 | 1180.78 | 2360.81 |

| Table S12. SARS-CoV-2 anti-S IgG titers among participants with subsequent SARS-CoV-2 infection. | | | | | | | | |
| --- | --- | --- | --- | --- | --- | --- | --- | --- |
| **Time point** | **SARS-CoV-2 infection^a^** | **N** | **Mean** | **Minimum** | **1st Quartile** | **Median** | **3rd Quartile** | **Maximum** |
| Post-V1 | No | 158 | 470.50 | 0.30 | 6.10 | 42.30 | 244.00 | 2501.00 |
|  | Yes | 3 | 1203.85 | 1090.00 | 1090.00 | 1155.05 | 1366.50 | 1366.50 |
| Post-V2 | No | 166 | 1087.61 | 0.20 | 20.20 | 364.00 | 2501.00 | 2501.00 |
|  | Yes | 4 | 1735.80 | 0.20 | 970.60 | 2221.00 | 2501.00 | 2501.00 |
| Post-V3 | No | 102 | 1877.43 | 0.70 | 817.00 | 2501.00 | 2501.00 | 2501.00 |
|  | Yes | 4 | 2501.00 | 2501.00 | 2501.00 | 2501.00 | 2501.00 | 2501.00 |
| End-of-study | No | 132 | 1625.25 | 0.20 | 184.50 | 2501.00 | 2501.00 | 2501.00 |
|  | Yes | 5 | 1199.28 | 0.20 | 0.20 | 994.00 | 2501.00 | 2501.00 |
| ^a^Participants are categorized based on whether they had a reported infection with SARS-CoV-2 after the first vaccine administration and before the next time point. | | | | | | | | |

| Table S13. Adjusted logistic regression models of the association of baseline and time-dependent variables with an observed positive SARS-CoV-2 anti-S IgG titer, *using vaccine timing as a categorical variable.* | | | | | |
| --- | --- | --- | --- | --- | --- |
| **Variable^a^** | **Category** | **N** | **Odds Ratio** | **99% CI** | **p-value** |
| **Post-V2 time point** | | | | | |
| Vaccine timing cohort^b^ | < 4 Months | 74 | 1.00 | - |  |
|  | 4-12 Months | 96 | 0.59 | (0.17, 1.20) | 0.04 |
| SARS-CoV-2 infection pre-enrollment | No | 153 | 1.00 | - |  |
|  | Yes | 17 | 3.16 | (0.86, 18.77) | 0.02 |
| Recipient SARS-CoV-2 vaccination pre-enrollment | No | 144 | 1.00 | - |  |
|  | Yes | 26 | 3.43 | (1.15, 16.02) | 0.004 |
| CD19+ B-Cell Count | ≤ Median^b^ | 59 | 1.00 | - |  |
|  | > Median | 63 | 3.29 | (1.04, 10.43) | 0.008 |
|  | Not measured | 48 | 4.16 | (0.98, 17.65) | 0.01 |
| CD4+ T-Cell Count | ≤ Median^b^ | 61 | 1.00 | - |  |
|  | > Median | 61 | 3.09 | (0.97, 9.84) | 0.01 |
|  | Not measured | 48 | - | - |  |
|  | | | | | |
| **Post-V3 time point** | | | | | |
| Vaccine timing cohort^b^ | < 4 Months | 72 | 1.00 | - |  |
|  | 4-12 Months | 94 | 0.69 | (0.30, 1.58) | 0.25 |
| SARS-CoV-2 infection pre-enrollment | No | 150 | 1.00 | - |  |
|  | Yes | 16 | 0.25 | (0.05, 1.41) | 0.04 |
|  | | | | | |
| **End-of-study time point** | | | | | |
| Vaccine timing cohort^b^ | < 4 Months | 57 | 1.00 | - |  |
|  | 4-12 Months | 80 | 1.06 | (0.42, 2.66) | 0.86 |
| Age (years) |  | 137 | 0.97 | (0.95, 1.00) | 0.03 |
| N indicates number; CI indicates confidence interval.  A stepwise variable selection procedure was used to determine covariates to include in the model, with a p-value <0.05 as the criterion for inclusion. Vaccine timing cohort was forced into the model. For each outcome, the displayed variables were the only variables retained in the adjusted model.  ^a^Covariates considered in models included baseline and time dependent variables from Tables 1 and 2. Time-dependent variables were considered in models at baseline as well as at the time point preceding each endpoint. Baseline variables were age, sex, race, ethnicity, underlying disease, graft source, donor and HLA match, conditioning intensity, GVHD prophylaxis, anti-Nucleocapsid IgG, calendar date of HCT, treatment center, and donor or recipient vaccination pre-enrollment. Time dependent variables were timing of vaccination, absolute lymphocyte count, absolute CD19+ B cell count, absolute CD4+ T cell count, receipt of Evusheld, receipt of IVIG, acute GVHD, chronic GVHD, immunosuppressive medication use, and recipient infection with SARS-CoV-2.  ^b^When vaccine timing cohorts were considered as <6 versus 6-12 months, the selected variables were the same with similar findings.  For immunosuppressive medications, corticosteroids were separately considered as a time-dependent variable at baseline as well as at the time point preceding each endpoint. | | | | | |

| Table S14. Adjusted logistic regression models of the association of baseline and time-dependent variables with an observed positive SARS-CoV-2 anti-S IgG titer, *using vaccine timing as a continuous variable.* | | | | | |
| --- | --- | --- | --- | --- | --- |
| **Variable^a^** | **Category** | **N** | **Odds Ratio** | **99% CI** | **p-value** |
| **Post-V2 time point** | | | | | |
| Vaccine timing post-HCT (months) |  | 170 | 0.86 | (0.67, 1.10) | 0.10 |
| SARS-CoV-2 infection pre-enrollment | No | 153 | 1.00 | - |  |
|  | Yes | 17 | 4.07 | (0.89, 18.55) | 0.02 |
| Recipient SARS-CoV-2 vaccination pre-HCT | No | 144 | 1.00 | - |  |
|  | Yes | 26 | 4.18 | (1.13, 15.50) | 0.005 |
| CD19+ B-Cell Count | ≤ Median^b^ | 59 | 1.00 | - |  |
|  | > Median | 63 | 3.54 | (1.11, 11.26) | 0.005 |
|  | Not measured | 48 | 4.19 | (0.99, 17.78) | 0.01 |
| CD4+ T-Cell Count | ≤ Median^b^ | 61 | 1.00 | - |  |
|  | > Median | 61 | 2.87 | (0.92, 8.91) | 0.02 |
|  | Not measured | 48 | - | - |  |
|  | | | | | |
| **Post-V3 time point** | | | | | |
| Vaccine timing post-HCT (months) |  | 166 | 1.03 | (0.85, 1.25) | 0.73 |
| SARS-CoV-2 infection pre-enrollment | No | 150 | 1.00 | - |  |
|  | Yes | 16 | 0.26 | (0.05, 1.45) | 0.04 |
|  | | | | | |
| **End-of-study time point** | | | | | |
| Vaccine timing post-HCT (months) |  | 137 | 1.02 | (0.82, 1.27) | 0.80 |
| Age (years) |  | 137 | 0.98 | (0.95, 1.01) | 0.03 |
| N indicates number; CI indicates confidence interval.  A stepwise variable selection procedure was used to determine covariates to include in the model, with a p-value <0.05 as the criterion for inclusion. Vaccine timing as a continuous variable was forced into the model. For each outcome, the displayed variables were the only variables retained in the adjusted model.  ^a^ Covariates considered in models included baseline and time dependent variables from Tables 1 and 2. Time-dependent variables were considered in models at baseline as well as at the time point preceding each endpoint. Baseline variables were age, sex, race, ethnicity, underlying disease, graft source, donor and HLA match, conditioning intensity, GVHD prophylaxis, anti-Nucleocapsid IgG, calendar date of HCT, treatment center, and donor or recipient vaccination pre-enrollment. Time dependent variables were timing of vaccination, absolute lymphocyte count, absolute CD19+ B cell count, absolute CD4+ T cell count, receipt of Evusheld, receipt of IVIG, acute GVHD, chronic GVHD, immunosuppressive medication use, and recipient infection with SARS-CoV-2.  For immunosuppressive medications, corticosteroids were separately considered as a time-dependent variable at baseline as well as at the time point preceding each endpoint. | | | | | |

| **Table S15.** Antibodies used for flow cytometry. | | |
| --- | --- | --- |
| Specificity | **Clone** | **Fluorophore** |
| CD69 | FN50 | BV750 |
| amine-reactive | n/a | LIVE/DEAD Blue |
| CD45RA | 5H9 | BUV395 |
| CD14 | MφP9 | BUV563 |
| CD45RO | UCHL1 | BUV805 |
| CD8 | 3B5 | Qdot800 |
| CD19 | SJ25-C1 | Qdot 605 |
| CD11c | B-ly6 | BUV661 |
| HLA-DR | L243 | BV570 |
| CD45 | 2D1 | PerCP |
| CD4 | SK3 | APC Fire 810 |
| CD3 | OKT3 | BV510 |

| Table S16: Power to Detect a 25% Difference in Immunogenicity Rates between 6-12 and <6 Month Cohorts with 41 and 112 Evaluable Patients (*a priori*). | |
| --- | --- |
| **Difference in Response Rates (6-12 mos. vs. < 6 mos.)** | **Power** |
| 90% vs. 65% or 10% vs. 35% | 92.8% |
| 80% vs. 55% or 20% vs. 45% | 85.4% |
| 70% vs. 45% or 30% vs. 55% | 81.7% |
| 60% vs. 35% or 40% vs. 65% | 81.6% |

| Table S17: Power to Detect a 25% Difference in Immunogenicity Rates between 4-12 and <4 Month Cohorts with 96 and 74 Evaluable Patients (*post-hoc*). | |
| --- | --- |
| **Difference in Response Rates (4-12 mos. vs. < 4 mos.)** | **Power** |
| 90% vs. 65% or 10% vs. 35% | 97.7% |
| 80% vs. 55% or 20% vs. 45% | 94.0% |
| 70% vs. 45% or 30% vs. 55% | 91.3% |
| 60% vs. 35% or 40% vs. 65% | 90.6% |

# REFERENCES

1. Huang Y, Borisov O, Kee JJ, et al. Calibration of two validated SARS-CoV-2 pseudovirus neutralization assays for COVID-19 vaccine evaluation. Sci Rep [Internet] 2021;11(1):1–13. Available from: https://doi.org/10.1038/s41598-021-03154-6

2. Van Gassen S, Callebaut B, Van Helden MJ, et al. FlowSOM: Using self-organizing maps for visualization and interpretation of cytometry data. Cytom Part A 2015;87(7):636–45.

3. Amir E ad D, Lee B, Badoual P, et al. Development of a comprehensive antibody staining database using a standardized analytics pipeline. Front Immunol 2019;10(JUN):1315.

4. Finak G, Langweiler M, Jaimes M, et al. Standardizing Flow Cytometry Immunophenotyping Analysis from the Human ImmunoPhenotyping Consortium. Sci Rep [Internet] 2016 [cited 2022 Nov 21];6. Available from: https://pubmed.ncbi.nlm.nih.gov/26861911/

5. Maecker HT, McCoy JP, Nussenblatt R. Standardizing immunophenotyping for the Human Immunology Project. Nat Rev Immunol 2012 123 [Internet] 2012 [cited 2022 Nov 21];12(3):191–200. Available from: https://www.nature.com/articles/nri3158
